# Differential Production of Type I IFN Determines the Reciprocal Levels of IL-10 and Proinflammatory Cytokines Produced by C57BL/6 and BALB/c Macrophages

**DOI:** 10.4049/jimmunol.1501923

**Published:** 2016-08-22

**Authors:** Ashleigh Howes, Christina Taubert, Simon Blankley, Natasha Spink, Xuemei Wu, Christine M. Graham, Jiawen Zhao, Margarida Saraiva, Paola Ricciardi-Castagnoli, Gregory J. Bancroft, Anne O’Garra

**Affiliations:** * Laboratory of Immunoregulation and Infection, The Francis Crick Institute, Mill Hill Laboratory, London NW7 1AA, United Kingdom;; † London School of Hygiene and Tropical Medicine, London WC1E 7HT, United Kingdom;; ‡ Department of Research and Innovation, Neo-Life Stem Cell Biotech Inc., Sichuan Umbilical Cord Blood Bank, Chengdu, Sichuan 610036, People’s Republic of China;; § Life and Health Sciences Research Institute, School of Health Sciences, University of Minho, 4710-057 Braga, Portugal;; ¶ Life and Health Sciences Research Institute, Biomaterials, Biodegradables and Biomimetics, Portuguese Government Associate Laboratory, 4710-057 Braga/Guimarães, Portugal;; ‖ Singapore Immunology Network, Agency for Science, Technology and Research, Singapore 138632, Singapore; and; # Department of Medicine, National Heart and Lung Institute, Imperial College London, London SW3 6LY, United Kingdom

## Abstract

Pattern recognition receptors detect microbial products and induce cytokines, which shape the immunological response. IL-12, TNF-α, and IL-1β are proinflammatory cytokines, which are essential for resistance against infection, but when produced at high levels they may contribute to immunopathology. In contrast, IL-10 is an immunosuppressive cytokine, which dampens proinflammatory responses, but it can also lead to defective pathogen clearance. The regulation of these cytokines is therefore central to the generation of an effective but balanced immune response. In this study, we show that macrophages derived from C57BL/6 mice produce low levels of IL-12, TNF-α, and IL-1β, but high levels of IL-10, in response to TLR4 and TLR2 ligands LPS and Pam3CSK4, as well as *Burkholderia pseudomallei*, a Gram-negative bacterium that activates TLR2/4. In contrast, macrophages derived from BALB/c mice show a reciprocal pattern of cytokine production. Differential production of IL-10 in *B. pseudomallei* and LPS-stimulated C57BL/6 and BALB/c macrophages was due to a type I IFN and ERK1/2-dependent, but IL-27–independent, mechanism. Enhanced type I IFN expression in LPS-stimulated C57BL/6 macrophages was accompanied by increased STAT1 and IFN regulatory factor 3 activation. Furthermore, type I IFN contributed to differential IL-1β and IL-12 production in *B. pseudomallei* and LPS-stimulated C57BL/6 and BALB/c macrophages via both IL-10–dependent and –independent mechanisms. These findings highlight key pathways responsible for the regulation of pro- and anti-inflammatory cytokines in macrophages and reveal how they may differ according to the genetic background of the host.

## Introduction

Proinflammatory immune responses are critical in the defense against pathogens; however, excessive inflammation has the potential to cause damage to the host. Thus, immunoregulatory pathways controlling inflammatory cytokine production are critical for ensuring an effective but balanced immune response (1). Pattern recognition receptor (PRR)–activated macrophages are an important early source of proinflammatory cytokines such as IL-12, TNF-α, and IL-1β, and their production is modulated by a complex array of direct and indirect regulatory mechanisms (1, 2).

A central negative regulator of inflammatory responses is the immunosuppressive cytokine IL-10 (3). The spontaneous onset of colitis in response to commensal gut flora in IL-10–deficient mice (4) and enhanced susceptibility of IL-10–deficient mice to septic shock (5) demonstrate the importance of IL-10 as a negative feedback regulator in the immune system. IL-10 can be produced by several cell types within the immune system, including PRR-stimulated macrophages (6), to which IL-10 can signal back in an autocrine manner and inhibit the production of proinflammatory cytokines via a STAT3-dependent mechanism (7–9).

Type I IFNs constitute a group of cytokines including IFN-β and multiple IFN-α proteins (10). Studies of the functions of type I IFN have revealed complex immunoregulatory roles for these cytokines. For example, type I IFN has been shown to promote the production of IL-10 from murine macrophages (11), human monocytes (12), and human dendritic cells (13), although the mechanisms by which this occurs are not fully understood. In the context of proinflammatory cytokine production, the effects of type I IFN are diverse. Mechanisms whereby type I IFN regulates distinct proinflammatory cytokines are as yet unclear (14–19). The role of type I IFN in vivo in the context of infection and inflammation is complex (20). Type I IFN has been shown in different settings to contribute to control of the pathogen or, conversely, may regulate or exacerbate inflammatory pathologies (20).

C57BL/6 and BALB/c mice differ significantly in their immune responses, giving rise to distinct outcomes of infection, and they have provided robust models for studying susceptibility or resistance to various pathogens (21–23). *Burkholderia pseudomallei* is a Gram-negative bacterium and the causative agent of melioidosis, a major cause of sepsis and mortality in endemic regions of Southeast Asia and Northern Australia and is increasingly being reported across the tropics. There is no available vaccine, antibiotic treatment is prolonged and not always effective, and mortality rates in acute cases can approach 50% even with optimal clinical management (24). Both proinflammatory cytokines and IL-10 are found at high levels in the plasma of individuals with acute infection, and their concentrations can predict mortality (25). Infection with *B. pseudomallei* serves as an important clinical and experimental example of Gram-negative sepsis, and resistance to infection is genetically determined. Several studies have shown that BALB/c mice, even when infected at low dose, will develop acute disease and succumb much earlier than C57BL/6 mice, which conversely can establish a long-term chronic infection with this pathogen when infected with a low bacterial load (26–28). Relative susceptibility to *B. pseudomallei* infection has been correlated with distinct profiles of proinflammatory cytokines produced by innate cells in addition to IFN-γ in *B. pseudomallei*–infected C57BL/6 and BALB/c mice (28–31). However, the complexities of in vivo infection models have made it difficult to fully dissect the mechanisms underlying differential cytokine production between these two strains of mice. Similarly, in the context of colitis, a disease associated with the elevated production of several proinflammatory cytokines, IL-10–deficient BALB/c mice are more susceptible than IL-10–deficient C57BL/6 mice (32), but again the mechanisms underlying this phenotype are incompletely understood. Thus, the in vitro study of cellular immune responses from these mice provides a valuable comparative model for the mechanistic dissection of cytokine regulation, which additionally may contribute to differences in resistance of C57BL/6 and BALB/c mice to infection and inflammatory diseases.

We report in the present study reciprocal profiles of IL-10 versus IL-12, TNF-α, and IL-1β production from C57BL/6 and BALB/c macrophages stimulated with *B. pseudomallei* and purified TLR2 and TLR4 ligands. Our investigation into these phenotypes revealed type I IFN to be a central mediator of differential cytokine production in C57BL/6 and BALB/c macrophages. Enhanced type I IFN production accounted for the reduced levels of IL-1β and IL-12 observed in C57BL/6 as compared with BALB/c macrophages, by IL-10–dependent and –independent mechanisms. We also show that prolonged IL-10 expression in C57BL/6 macrophages results from type I IFN–induced ERK1/2 activation. These results support an important immunoregulatory role for type I IFN together with IL-10 and demonstrate that this activity is dependent on the genetic background of the host.

## Materials and Methods

### Animals

C57BL/6 wild-type (WT), BALB/c WT, C57BL/6 *Il10^−/−^*, BALB/c *Il10^−/−^*, and all other mutant mice were bred and maintained at The Francis Crick Institute, Mill Hill Laboratory under specific pathogen-free conditions in accordance with the Home Office, U.K., Animal Scientific Procedures Act, 1986. *Tlr4*^−/−^ and *Trif*^−/−^ breeding pairs, all on a C57BL/6 background, were provided by Prof. S. Akira (Osaka University, Osaka, Japan). C57BL/6 *Ifnar1^−/−^* breeders originated from B&K Universal (Hull, U.K.), and C57BL/6 *Tccr^−/−^* (referred to as *Il27ra^−/−^* in text) breeders were provided by Genentech (South San Francisco, CA). All mice used were females between 8 and 16 wk of age.

### Generation and stimulation of bone marrow–derived macrophages

Bone marrow–derived macrophages (BMDMs) were generated as previously described (33). On day 6, adherent cells were harvested and seeded in 48-well tissue culture plates at 0.5 × 10^6^ cells/well and rested for 18–20 h prior to stimulation. Cells were stimulated, unless otherwise stated, with 10 ng/ml *Salmonella minnesota* LPS (Alexis Biochemicals), 200 ng/ml Pam3CSK4 (InvivoGen), or heat-killed (to avoid heavy Containment Level 3 work) *B. pseudomallei* 576 at a ratio of 5–500 *B. pseudomallei* to 1 BMDM. Data were verified to be similar at the cytokine protein and mRNA levels using live *B. pseudomallei* 576 (data not shown). When indicated, cells were treated with rIFN-β (PBL) or rIL-27 (R&D Systems), both of which were shown to have very low levels of <1 endotoxin unit/μg endotoxin only, which on dilution in LPS-free media for assay resulted in <0.02 endotoxin unit/ml. Abs, 10 μg/ml anti-IFNAR1 mAb (clone MAR1-5A3, mouse IgG1; Bio X Cell), 10 μg/ml anti–IL-10R (clone 1B1.3a, rat IgG1), or relevant isotype control (clones GL113 or TC31.2F11, respectively) were all gifts from DNAX Research Institute (now Merck, Palo Alto, CA). MEK inhibitor PD184352 (1 μM) or PD0325901 (0.1 μM) and p38 inhibitor SB203580 (0.5 μM) were added to BMDMs 1 h prior or 2 h after stimulation with LPS as indicated (34).

### Cytokine quantification

Cytokines were quantified from supernatants of stimulated cells. IL-10 and IL-12p40 were quantified by ELISA. Cone JES5-2A5 (eBioscience) was used for IL-10 capture, and biotinylated anti-mouse IL-10 SXC-1 (BD Biosciences) was used for detection. Clone C15.6.7 was used for IL-12p40 capture, and biotinylated anti-mouse IL-12p40 C17.8 was used for detection (both gifts from DNAX Research Institute), followed by HRP-conjugated streptavidin (Jackson ImmunoResearch Laboratories). IL-12p70, TNF-α, and IL-27 (eBioscience), IL-1β (R&D Systems), and IFN-β (PBL) were quantified using commercially available ELISA kits.

### RNA isolation and quantitative real-time PCR

RNA was harvested and isolated using an RNeasy mini kit (Qiagen) according to the manufacturer’s instructions. cDNA was synthesized using a high-capacity cDNA reverse transcription kit (Applied Biosystems), according to the manufacturer’s instructions, followed by RNase H (Promega) treatment for 30 min at 37°C. *Il10*, *Il12a*, *Ifnb1*, *Oas1g*, *Stat1*, *Stat3*, *Irf7*, *Irf9*, and *Tlr4* gene expression were quantified by quantitative real-time PCR (qRT-PCR; 7900HT; Applied Biosystems) using the TaqMan system, and normalized to *Hprt1* mRNA. Primer probes used were *Il10* (Mm00439616_m1), *Il12a* (Mm00434165_m1), *Ifnb1* (Mm00439552_s1); *Oas1g* (Mm01730198_m1), *Stat1* (Mm_00439518_m1), *Stat3* (Mm_01219775_m1), *Irf7* (Mm_00516793_g1), *Irf9* (Mm_00492679_m1), *Tlr4* (Mm0045273_m1), and *Hprt1* (Mm00446968_m1), all purchased from Applied Biosystems. For the quantification of premature *Il10* mRNA, the following primers were designed using Primer Express 2.0 software and custom made by Applied Biosystems: forward (exon 3), 5′-AGCATGGCCCAGAAATCAAG-3′; probe (exon 3), 5′-CTCAGGATGCGGCTGA-3′; reverse (intron 4), 5′-AGAACGCATCTGCTACTCACACA-3′.

### FACS staining

For FACS analysis, BMDMs were stimulated with LPS, washed, and blocked with anti-CD16/CD32 Ab. Cells were then stained with PE-labeled anti-mouse TLR4 (SA15-21; BioLegend) for 30 min at 4°C and acquired using a BD LSR II (BD Biosciences). Data were analyzed by FlowJo software.

### Microarray processing and analysis

RNA quality was confirmed (RNA integrity number, range 9–10) using an Agilent 2100 Bioanalyzer (Agilent Technologies). RNA was prepared for microarray analysis using the Illumina TotalPrep-96 RNA amplification kit following the manufacturer’s instructions. cRNA (1500 ng) was hybridized onto Illumina BeadChip arrays (MouseWG-6 v2) and scanned by an Illumina iScan. Signal intensity calculations and background subtraction were performed using GenomeStudio software (Illumina). Analyses of microarray data were done using GeneSpring GX software version 12.6.1 (Agilent Technologies). A lower threshold of signal intensity was set to 10, and the expression values were log transformed (base2) and scaled to the 75th percentile for normalization. The expression value of each gene probe was then normalized to the median of expression of that gene probe in all samples. Gene probes were quality filtered for those present (*p* < 0.01) in at least one sample, with 19,191 gene probes having remained. Further statistical analysis and generation of gene lists are described in the relevant figure legends. Canonical pathway analysis was conducted using Ingenuity Pathway Analysis (IPA) software (Ingenuity Systems, http://www.ingenuity.com). Expression data conform to the minimum information about a microarray experiment standards for microarray analysis. Microarray data have been deposited in the Gene Expression Omnibus database (http://www.ncbi.nlm.nih.gov/geo/) under accession number GSE79809.

### Determination of mRNA stability

BMDMs were stimulated with LPS, and 1 h later 10 μg/ml actinomycin D (*Streptomyces* sp., Sigma-Aldrich) was added (*t* = 0) to the cultures. mRNA was harvested after 30, 60, or 90 min, reverse transcribed into cDNA, and quantified by qRT-PCR.

### Quantification of IFN regulator factor 3 activation

Nuclear extracts of 2 h heat-killed *B. pseudomallei*–stimulated BMDMs (5:1 *B. pseudomallei*/BMDM) were prepared with the Nuclear Extract Kit and assayed with the TransAM IFN regulatory factor (IRF) 3 kit (both from Active Motif) according to the manufacturer’s instructions.

### Western blotting

BMDMs were rested in 1% FCS for 20 h prior to stimulation with LPS or rIFN-β. At the indicated time points cells were washed with PBS and lysed in RIPA buffer as previously described (35) or in Triton X-100 lysis buffer (for IRF3 Western blots). Proteins were resolved on a 12.5% SDS-polyacrylamide gel and transferred to polyvinylidene difluoride membranes (Millipore). Membranes were probed with Abs against phospho-ERK1/2 (T185/Y187), total ERK1/2 (both Invitrogen), phospho-p38 (T180/Y182), total p38, phospho-STAT1 (Y701), total STAT1, phospho-IRF3 (S396, 4D4G) (Cell Signaling Technology), total IRF3, GAPDH (FL-335), heat shock protein 90α/β (H-114) (Santa Cruz Biotechnology), or actin (Calbiochem) followed by HRP-conjugated goat anti-rabbit IgG, rabbit anti-goat IgG (SouthernBiotech), or goat anti-mouse IgM (Calbiochem), and visualized using Pierce ECL Western blotting substrate (Thermo Scientific) or Luminata Crescendo Western chemiluminescent HRP substrate (Millipore). Western blots were quantified using Quantity One software.

### Statistical analysis

GraphPad Prism software was used to analyze data by one- or two-way ANOVA with Bonferroni multiple comparison testing or a Student *t* test. Statistical analyses of microarray data were done using GeneSpring GX software version 12.6.1 (Agilent Technologies) and are described in the relevant figure legends.

## Results

### *B. pseudomallei,* LPS, and Pam3CSK4 induce higher levels of IL-10 but lower levels of proinflammatory cytokines in C57BL/6 compared with BALB/c macrophages

**FIGURE 1.**
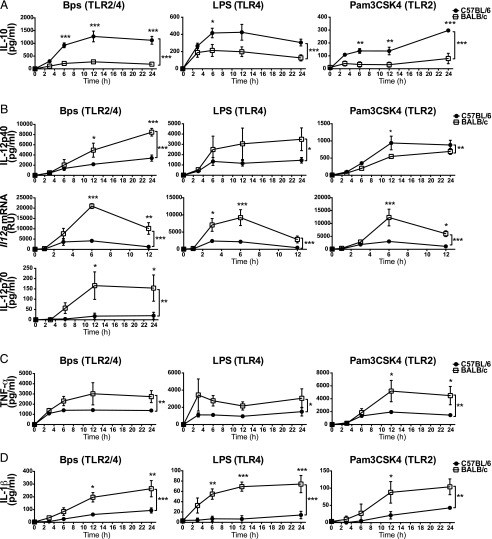
C57BL/6 macrophages produce higher levels of IL-10 but lower levels of proinflammatory cytokines compared with BALB/c macrophages in response to bacterial products. C57BL/6 and BALB/c BMDMs were stimulated with *B. pseudomallei*, LPS, or Pam3CSK4 for the indicated times. IL-10 (**A**), IL-12p40, IL-12p70 (**B**), TNF-α (**C**), and IL-1β (**D**) protein levels in supernatants were determined by ELISA. *Il12a* mRNA expression (B) was determined by qRT-PCR and normalized to *Hprt1* mRNA expression. Graphs show means ± SEM of three independent experiments. *p < 0.05, **p < 0.01, ***p < 0.001 as determined by two-way ANOVA (Bonferroni multiple comparison test). Bps, *B. pseudomallei.*

To better understand the factors regulating the production of pro- and anti-inflammatory cytokines in innate immune cells, we investigated TLR-induced IL-10, IL-12, TNF-α, and IL-1β production from C57BL/6 and BALB/c macrophages. C57BL/6 and BALB/c macrophages were stimulated over a time course with either *B. pseudomallei*, which activates both TLR2 and TLR4 signaling (36–38), or purified TLR2 and TLR4 ligands Pam3CSK4 and LPS, respectively (Fig. 1). Significantly higher levels of IL-10 were produced by C57BL/6 as compared with BALB/c macrophages with all three stimuli across the time course of stimulation (Fig. 1A). IL-12 is a heterodimeric cytokine composed of IL-12p40 and IL-12p35 subunits, which generate the biologically active IL-12p70 (39). In contrast to IL-10, levels of IL-12p40 production were higher in *B. pseudomallei*– and LPS-stimulated BALB/c compared with C57BL/6 macrophages (Fig. 1B). However, the levels of IL-12p40 production were marginally higher in C57BL/6 macrophages stimulated with Pam3CSK4 (Fig. 1B). The expression of *Il12a* mRNA (which encodes the IL-12p35 subunit) was significantly higher in BALB/c compared with C57BL/6 macrophages stimulated with *B. pseudomallei*, LPS, or Pam3CSK4 (Fig. 1B). Consistent with this, the level of IL-12p70 production was significantly higher in *B. pseudomallei*–stimulated BALB/c compared with C57BL/6 macrophages (Fig. 1B), although generally below the limit of detection in LPS- or Pam3CSK4-stimulated cells. We also observed higher levels of TNF-α (Fig. 1C) and IL-1β (Fig. 1D) production from BALB/c compared with C57BL/6 macrophages across the time course of *B. pseudomallei*, LPS, and Pam3CSK4 stimulation. Elevated levels of IL-10 but lower proinflammatory cytokine production were also observed in C57BL/6 relative to BALB/c macrophages when stimulated with a range of doses of *B. pseudomallei*, LPS, and Pam3CSK4 (Supplemental Fig. 1).

IL-10 has the ability to suppress proinflammatory cytokine production from macrophages (3). To investigate whether the low levels of IL-12, TNF-α, and IL-1β production from C57BL/6 compared with BALB/c macrophages were due to the much higher levels of IL-10 produced by C57BL/6 macrophages, *B. pseudomallei*–, LPS-, and Pam3CSK4-stimulated C57BL/6 *Il10^−/−^* and BALB/c *Il10^−/−^* macrophages were assessed for their levels of IL-12p70, TNF-α, and IL-1β production (Supplemental Fig. 2). IL-12p70 production was found to be greatly increased in *Il10^−/−^* macrophages relative to WT cells from both strains of mice; however, the levels remained higher in BALB/c compared with C57BL/6 macrophages in response to *B. pseudomallei* or LPS stimulation (Supplemental Fig. 2A). In Pam3CSK4-stimulated cells, the increase in IL-12p70 production in IL-10–deficient cells compared with WT was modest, although the levels of IL-12p70 production were the same in C57BL/6 *Il10^−/−^* and BALB/c *Il10^−/−^* macrophages (Supplemental Fig. 2A). In contrast, TNF-α production was equivalent in *B. pseudomallei–*, LPS-, and Pam3CSK4-stimulated C57BL/6 *Il10^−/−^* and BALB/c *Il10^−/−^* macrophages (Supplemental Fig. 2B). IL-1β production remained higher in *B. pseudomallei*– and LPS-stimulated BALB/c *Il10^−/−^* compared with C57BL/6 *Il10^−/−^* macrophages, but it was more comparable in Pam3CSK4-stimulated cells (Supplemental Fig. 2C). Thus, the differential production of TNF-α by all stimuli, as well as the differences in Pam3CSK4-induced IL-12p70 and IL-1β, was largely explained by IL-10–mediated inhibition. However, the differential production of IL-12p70 and IL-1β in *B. pseudomallei*– and LPS-stimulated C57BL/6 and BALB/c macrophages, although it may in part be explained by IL-10, was still observed in the complete absence of IL-10, suggesting an additional mechanism of inhibition in C57BL/6 macrophages. Additionally, the factors contributing to the differential production of IL-10 itself in C57BL/6 and BALB/c macrophages remained unclear.

### *B. pseudomallei*–stimulated C57BL/6 macrophages express higher levels of type I IFN pathway–related genes compared with BALB/c macrophages

**FIGURE 2.**
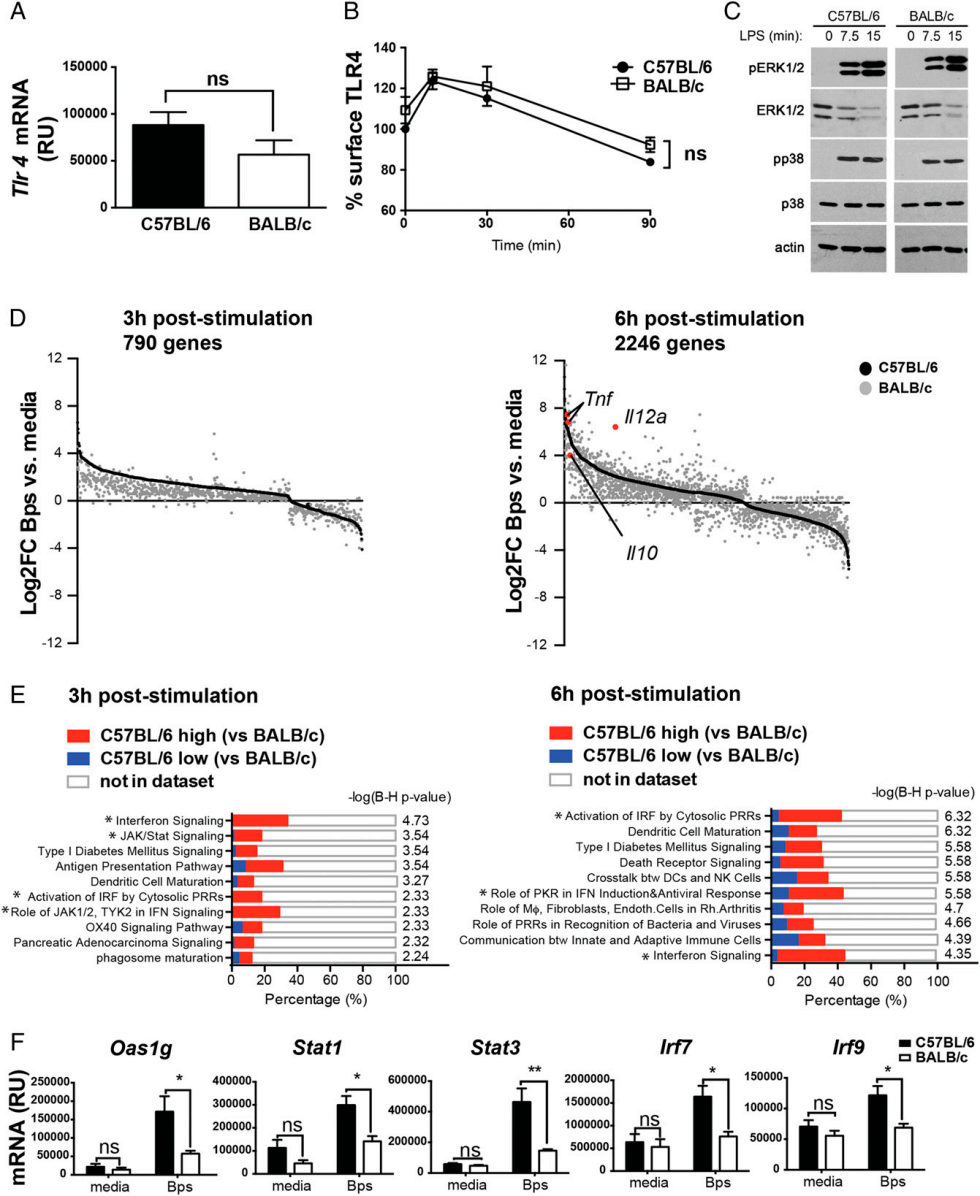
C57BL/6 macrophages show similar early TLR4-induced responses but higher expression of type I IFN pathway genes, as compared with BALB/c macrophages. (**A**) *Tlr4* mRNA expression in C57BL/6 and BALB/c BMDMs at steady-state was determined by qRT-PCR and normalized to *Hprt1* mRNA expression. (**B**) C57BL/6 and BALB/c BMDMs were stimulated with LPS for the indicated times and TLR4 expression was analyzed by flow cytometry. (**C**) BMDMs were stimulated with LPS as indicated, and phosphorylation of ERK1/2 and p38 in whole-cell lysates was determined by Western blotting. (**D** and **E**) C57BL/6 and BALB/c BMDMs were stimulated with *B. pseudomallei* for 3 or 6 h in triplicate cultures. Total RNA was isolated and processed for microarray analysis as described in *Materials and Methods*. (D) Genes significantly differentially regulated by *B. pseudomallei* in C57BL/6 and BALB/c BMDMs were identified by two-way ANOVA analysis (*p* < 0.01, Benjamini–Hochberg false discovery rate) and the selection of genes that were significantly different due to both stimulation and strain. Genes significantly changed due to stimulus alone or strain alone were excluded from the analysis. Expression level of individual genes (black dots, C57BL/6; gray dots, BALB/c) are shown as log_2_ fold change over corresponding media control samples and ordered according to their C57BL/6 expression level. (E) Top 10 IPA pathways significantly associated with the genes differentially regulated by *B. pseudomallei* stimulation in C57BL/6 and BALB/c macrophages at each time point are shown. The *x*-axis represents the percentage overlap between input genes and annotated genes within the pathway. Colors within bars denote genes that are more highly expressed in C57BL/6 or BALB/c macrophages. Benjamini–Hochberg (B-H) corrected −log(*p* value) represents pathway association. *Type I IFN–related pathway. (**F**) C57BL/6 and BALB/c BMDMs were stimulated with *B. pseudomallei* for 6 h. Gene expression was determined by qRT-PCR and expression values were normalized to *Hprt1* mRNA expression. All graphs show means ± SEM of three independent experiments. Western blot (C) is representative of two independent experiments.*p < 0.05, **p < 0.01, ***p < 0.001 as determined by two-way ANOVA (Bonferroni multiple comparison test).

To investigate further the potential mechanisms underlying differential production of IL-10 and proinflammatory cytokines by TLR4-stimulated C57BL/6 and BALB/c macrophages, we first undertook experiments to address whether this was attributable to differential TLR4 expression or early signaling events downstream of this receptor. Steady-state *Tlr4* mRNA expression, TLR4 surface expression, and TLR4 endocytosis post-LPS stimulation were the same in C57BL/6 and BALB/c macrophages (Fig. 2A, 2B). Early activation of the MAPKs ERK1/2 or p38 by LPS was also similar in C57BL/6 and BALB/c macrophages (Fig. 2C). These data suggest that the differential cytokine production in BALB/c versus C57BL/6 macrophages was not simply due to differential TLR abundance or immediate signaling downstream of TLR4.

We therefore undertook an unbiased microarray analysis of these cells stimulated with *B. pseudomallei* for 3 and 6 h. At 3 h, 790 genes were found to be differentially regulated by *B. pseudomallei* in C57BL/6 and BALB/c macrophages (Fig. 2D). Most of these genes were upregulated by *B. pseudomallei* stimulation in both strains of mice, and of these almost all were more strongly upregulated in C57BL/6 compared with BALB/c macrophages. The genes downregulated by *B. pseudomallei* stimulation in C57BL/6 macrophages were also downregulated in BALB/c macrophages, although either more strongly or more weakly in comparison. At 6 h, 2246 genes were found to be differentially regulated by *B. pseudomallei* in C57BL/6 and BALB/c macrophages (Fig. 2D), suggesting a reinforcement of differential gene expression over time. These genes included those up- and downregulated by *B. pseudomallei* stimulation and, in contrast to the 3 h time point, were either more strongly or weakly induced in BALB/c macrophages, resulting in a complex profile of gene expression (Fig. 2D). Of note, *Il10* (BALB/c low, gray dot highlighted red in Fig. 2D, 6 h after stimulation), *Il12a*, and *Tnf* (BALB/c high, gray dots highlighted red in Fig. 2D, 6 h after stimulation) were found to be present at similar relative levels between BALB/c and C57BL/6 macrophages, as observed at the protein level (Fig. 1). IL-1β was not found to be significantly differentially expressed at the mRNA level, suggesting that this cytokine may be differentially regulated posttranscriptionally in C57BL/6 and BALB/c macrophages.

To better understand the biological relationships between the differentially regulated genes, transcripts identified at 3 and 6 h were subjected to canonical pathway analysis (IPA). Among the top 10 pathways significantly associated with the gene lists at each time point, several were found to relate to type I IFN–mediated processes. These included IFN signaling, JAK/STAT signaling, activation of IRF by cytosolic PRRs, and role of JAK1, JAK2, TYK2 in IFN signaling at 3 h, and activation of IRF by cytosolic PRRs, role of PKR in IFN induction and antiviral response, and IFN signaling at 6 h (Fig. 2E, Supplemental Table I). Most of the differentially regulated genes within these type I IFN–related pathways, including *Oas1g*, *Stat1*, *Stat3*, *Irf7*, and *Isgf3g* (IRF9), were more highly expressed in C57BL/6 macrophages (Supplemental Table I), and this was validated by qRT-PCR (Fig. 2F). Additionally, the dominance of the IFN-inducible signaling pathway genes in C57BL/6 versus BALB/c macrophages was validated in an independent experiment using live *B. pseudomallei* (data not shown). These data suggest that enhanced type I IFN signaling in C57BL/6 compared with BALB/c macrophages may be a fundamental difference in the responses of these cells to *B. pseudomallei* and may explain their differential expression of IL-10 versus proinflammatory cytokines.

### BALB/c macrophages show reduced IFN-β production and STAT1 and IRF3 activation compared with C57BL/6 macrophages upon TLR4 stimulation

**FIGURE 3.**
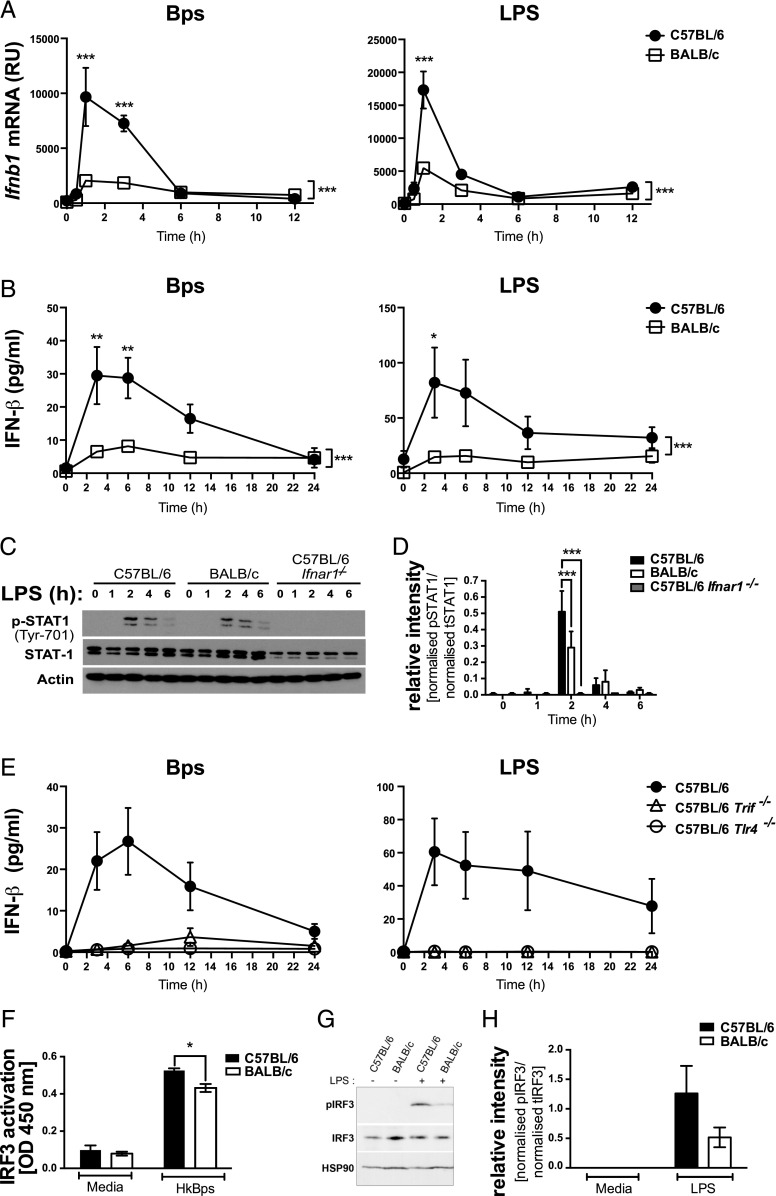
TLR4-dependent IFN-β production and STAT1 and IRF3 activation are higher in C57BL/6 compared with BALB/c macrophages. BMDMs were stimulated with *B. pseudomallei* or LPS for the indicated times. (**A**) *Ifnb1* mRNA expression was determined by qRT-PCR and normalized to *Hprt1* mRNA expression. (**B**) IFN-β production was quantified by ELISA. (**C**) Whole-protein extracts were generated and analyzed by Western blot for total and phosphorylated STAT1, and actin loading control. (**D**) Relative intensity of two independent experiments shown for data represented in (C). (**E**) IFN-β production was quantified by ELISA. (**F**) C57BL/6 and BALB/c macrophages were stimulated with *B. pseudomallei* for 2 h, and nuclear extracts were analyzed for active IRF3 by ELISA. (**G**) Whole-protein extracts were generated and analyzed by Western blot for total and phosphorylated IRF3 and heat shock protein 90 loading control. (**H**) Relative intensity of three independent experiments shown from data in (G). Graphs show means ± SEM of two to four (E) or at least three independent experiments (A and B). *p < 0.05, **p < 0.01, ***p < 0.001 as determined by two-way ANOVA (Bonferroni multiple comparison test).

In keeping with higher expression of type I IFN–inducible genes in C57BL/6 macrophages as shown by our microarray analysis (Fig. 2), *Ifnb1* mRNA was more highly expressed in *B. pseudomallei*– and LPS-stimulated C57BL/6 macrophages compared with BALB/c macrophages (Fig. 3A). In both strains, *Ifnb1* mRNA expression peaked at 1 h, returning to near baseline levels by 6 h, explaining why in the microarray analysis, which was conducted at later time points, *Ifnb1* itself was not identified as a C57BL/6-high gene (Supplemental Table I). Protein levels of IFN-β production, which peaked at 3 h, were also higher in *B. pseudomallei*– and LPS-stimulated C57BL/6 compared with BALB/c macrophages (Fig. 3B). We additionally assessed the phosphorylation of STAT1, activated downstream of the type I IFN receptor, in LPS-stimulated C57BL/6, BALB/c, and C57BL/6 *Ifnar1^−/−^* macrophages (Fig. 3C, 3D). STAT1 was phosphorylated in macrophages from both strains of mice but was reduced in BALB/c macrophages compared with C57BL/6 after 2 h of stimulation. Thus, the level of type I IFN produced by BALB/c macrophages is sufficient to activate STAT1, but not to the level seen in C57BL/6 macrophages. The total absence of STAT1 phosphorylation in LPS-stimulated C57BL/6 *Ifnar1^−/−^* macrophages confirmed that the STAT1 activation in WT macrophages was due to type I IFN signaling (Fig. 3C, 3D).

To investigate the mechanism by which *B. pseudomallei* induces IFN-β production, C57BL/6 *Trif^−/−^* and C57BL/6 *Tlr4^−/−^* macrophages were assessed for their production of IFN-β in response to *B. pseudomallei* or LPS (Fig. 3E). In the absence of TRIF or TLR4, neither *B. pseudomallei* nor LPS was able to induce IFN-β production, suggesting that differential production of this cytokine in *B. pseudomallei*– or LPS-stimulated C57BL/6 and BALB/c macrophages may be a result of differential activation of the TLR4/TRIF pathway. In support of this, the activation of IRF3, which is required for IFN-β production downstream of TLR4 (40), was lower in *B. pseudomallei*–stimulated BALB/c macrophages (Fig. 3F–H).

### Type I IFN signaling mediates the higher level of IL-10 production observed in *B. pseudomallei–* and LPS-stimulated C57BL/6 compared with BALB/c macrophages

**FIGURE 4.**
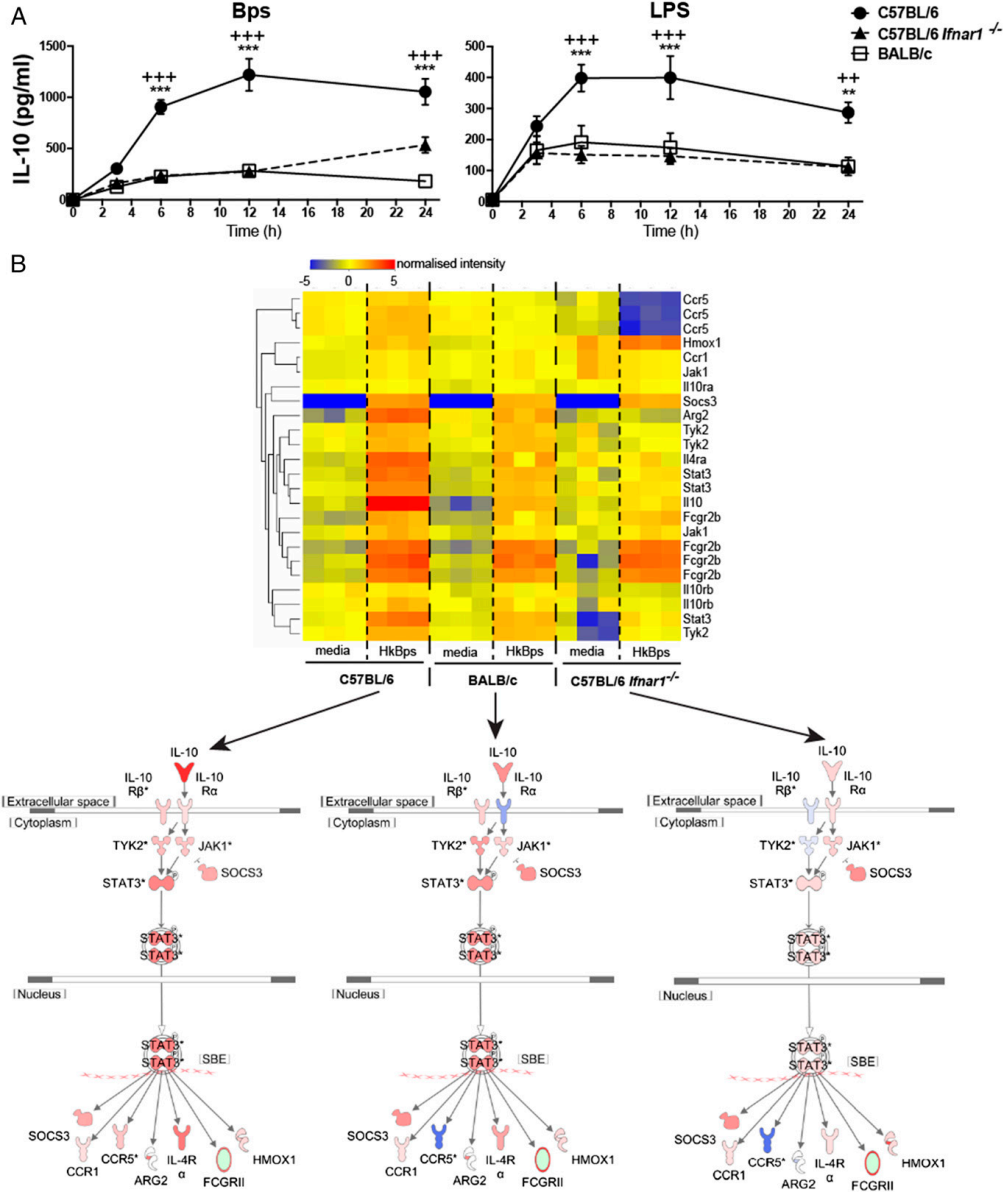
*B. pseudomallei*– and LPS-stimulated C57BL/6 *Ifnar1^−/−^* and BALB/c macrophages have similar levels of IL-10 production and show reduced expression of IL-10 pathway genes compared with C57BL/6 WT macrophages. (**A**) C57BL/6, BALB/c, and C57BL/6 *Ifnar1^−/−^* BMDMs were stimulated with *B. pseudomallei* or LPS for the indicated times. IL-10 production was quantified by ELISA. Graphs show means ± SEM of four independent experiments. **p < 0.01, ***p < 0.001 as determined by two-way ANOVA (*C57BL/6 versus BALB/c; ^+^C57BL/6 versus C57BL/6 *Ifnar1^−/−^*), Bonferroni multiple comparison test. (**B**) C57BL/6, BALB/c, and C57BL/6 *Ifnar1^−/−^* BMDMs were stimulated with *B. pseudomallei* for 6 h in triplicate cultures. Total RNA was isolated and processed for microarray analysis as described in *Materials and Methods*. Normalized expression of genes annotated within the IL-10 pathway (IPA) is shown hierarchically clustered according to expression. Lower panel shows overlay of gene expression level (red high, blue low) on a schematic of the IL-10 pathway (IPA).

Type I IFN has been reported to positively regulate IL-10 production in macrophages stimulated with LPS or infected with bacteria such as *Listeria monocytogenes* or *Mycobacterium tuberculosis* (11, 16, 33, 41, 42). To determine whether autocrine type I IFN signaling mediated the higher level of IL-10 production in C57BL/6 compared with BALB/c macrophages, C57BL/6 *Ifnar1^−/−^* macrophages were stimulated with *B. pseudomallei* or LPS over a time course. The level of IL-10 production was significantly reduced in C57BL/6 *Ifnar1^−/−^* macrophages compared with C57BL/6 WT macrophages, and it was reduced to the level of IL-10 produced by BALB/c macrophages (Fig. 4A). Microarray analysis of C57BL/6 WT, BALB/c, and C57BL/6 *Ifnar1^−/−^* macrophages stimulated with *B. pseudomallei* for 6 h revealed lower expression of *Il10* mRNA and IL-10 pathway genes, including *Stat3*, *Jak1*, *Ccr5*, *Il4ra*, and *Arg2*, in BALB/c and C57BL/6 *Ifnar1^−/−^* macrophages relative to C57BL/6 WT macrophages (Fig. 4B). These data support a role for type I IFN in the differential regulation of IL-10 in *B. pseudomallei*– and LPS-stimulated C57BL/6 and BALB/c macrophages.

### Type I IFN signaling promotes IL-10 through active transcription and stabilization of *Il10* mRNA

**FIGURE 5.**
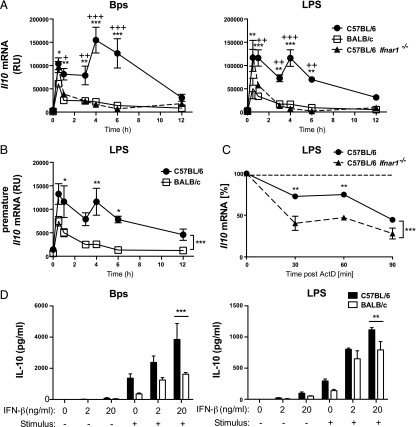
Type I IFN promotes *B. pseudomallei*– and LPS-stimulated IL-10 production transcriptionally and by stabilization of *Il10* mRNA in C57BL/6 and BALB/c macrophages. (**A** and **B**) C57BL/6, BALB/c, and C57BL/6 *Ifnar1^−/−^* BMDMs were stimulated with *B. pseudomallei* or LPS for the indicated times. (**C**) C57BL/6 and C57BL/6 *Ifnar1^−/−^* BMDMs were stimulated with LPS for 1 h and treated with actinomycin D (ActD). (A–C) *Il10* mRNA was harvested and quantified by qRT-PCR, normalized to *Hprt1* mRNA levels. (**D**) C57BL/6 and BALB/c BMDMs were treated with 2 or 20 ng/ml IFN-β for 2 h prior to stimulation with *B. pseudomallei* or LPS for 24 h. IL-10 production was quantified by ELISA. Graphs show means ± SEM of two to three independent experiments. *p < 0.05, **p < 0.01, ***p < 0.001 by two-way ANOVA (*C57BL/6 versus BALB/c, ^+^C57BL/6 versus C57BL/6 *Ifnar1^−/−^*), Bonferroni multiple comparison test.

To investigate the mechanisms by which type I IFN promotes IL-10 production in C57BL/6 macrophages, a detailed time course of *Il10* mRNA kinetics was carried out on *B. pseudomallei*– and LPS-stimulated C57BL/6 WT, BALB/c, and C57BL/6 *Ifnar1^−/−^* macrophages. *B. pseudomallei*– and LPS-stimulated C57BL/6 WT macrophages expressed an initial *Il10* mRNA peak at 0.5 h followed by a second peak at 4 h (Fig. 5A). BALB/c macrophages expressed the initial peak of *Il10* mRNA, although at a lower magnitude relative to C57BL/6 WT macrophages; however, they completely lacked the second peak of *Il10* mRNA (Fig. 5A). In C57BL/6 *Ifnar1^−/−^* macrophages, although the first peak of *Il10* mRNA was mostly unaffected by the absence of type I IFN signaling, the second peak was completely abrogated (Fig. 5A). This suggests that autocrine type I IFN activates a late transcriptional wave of *Il10* mRNA expression, supported by the presence of a second peak of premature *Il10* mRNA, indicative of active transcription, in LPS-stimulated C57BL/6 but not BALB/c macrophages (Fig. 5B). Additionally, whereas in BALB/c and C57BL/6 *Ifnar1^−/−^* macrophages a rapid decay of the *Il10* mRNA is observed after 0.5 h, in C57BL/6 macrophages this decay is much less pronounced (Fig. 5A). Thus, we further investigated whether there was an additional effect of type I IFN on *Il10* mRNA stability. We observed reduced *Il10* mRNA stability in C57BL/6 *Ifnar1^−/−^* macrophages compared with C57BL/6 WT macrophages, demonstrating that autocrine type I IFN also has a stabilizing effect on *Il10* mRNA (Fig. 5C).

It has been reported that in LPS-stimulated macrophages, type I IFN–induced IL-27 is required for the optimal enhancement of IL-10 by type I IFN (43). However, others have shown that murine macrophages stimulated with LPS are unresponsive to IL-27 (44). To investigate whether the type I IFN–mediated enhancement of IL-10 production in C57BL/6 WT macrophages is dependent on IL-27, C57BL/6, BALB/c, and C57BL/6 *Ifnar1^−/−^* macrophages were stimulated with *B. pseudomallei* or LPS and IL-27 production was determined (Supplemental Fig. 3A). In response to both stimuli, the overall magnitude of IL-27 production was similar in C57BL/6 and BALB/c macrophages. In C57BL/6 *Ifnar1^−/−^* macrophages, IL-27 production was drastically reduced (Supplemental Fig. 3A), in agreement with previous studies (45) showing a role for type I IFN in the promotion of IL-27 in C57BL/6 macrophages. However, *B. pseudomallei*– and LPS-stimulated C57BL/6 *Il27ra^−/−^* macrophages revealed no significant difference in IL-10 production as compared with WT macrophages (Supplemental Fig. 3B). Additionally, IL-10 production was similarly enhanced by addition of exogenous IFN-β in WT C57BL/6 and C57BL/6 *Il27ra^−/−^* macrophages (Supplemental Fig. 3C). Furthermore, the addition of exogenous IL-27 did not enhance IL-10 production by LPS-stimulated C57BL/6, BALB/c, or C57BL/6 *Ifnar1^−/−^* macrophages (Supplemental Fig. 3D). These data demonstrate that type I IFN–mediated enhancement of IL-10 in *B. pseudomallei*– and LPS-stimulated C57BL/6 macrophages is independent of IL-27.

### Addition of type I IFN enhances IL-10 production in both C57BL/6 and BALB/c macrophages stimulated with *B. pseudomallei* and LPS

To test whether the addition of type I IFN could enhance IL-10 production in BALB/c macrophages and potentially rescue IL-10 production in this strain, we treated C57BL/6 and BALB/c macrophages with exogenous IFN-β in the presence or absence of PRR ligation by *B. pseudomallei* or LPS (Fig. 5D). Treatment with IFN-β in the absence of PRR stimulation did not induce IL-10 production from C57BL/6 or BALB/c macrophages. In *B. pseudomallei*–stimulated cells, the addition of IFN-β greatly enhanced IL-10 production in C57BL/6 and BALB/c macrophages; however, IL-10 production remained significantly higher in C57BL/6 macrophages (Fig. 5D). In LPS-stimulated macrophages, the addition of IFN-β also enhanced IL-10 production in both C57BL/6 and BALB/c macrophages (Fig. 5D). Furthermore, upon treatment with IFN-β, the levels of LPS-induced IL-10 from BALB/c macrophages were as high as those from C57BL/6 macrophages (Fig. 5D). BALB/c macrophages therefore have the capacity to produce enhanced levels of IL-10 in response to IFN-β treatment. Furthermore, in LPS-stimulated cells, IFN-β had the potential to fully restore IL-10 production to the level observed in similarly stimulated C57BL/6 macrophages.

### Type I IFN stimulates IL-10 production in C57BL/6 macrophages through the activation of ERK1/2 MAPK

**FIGURE 6.**
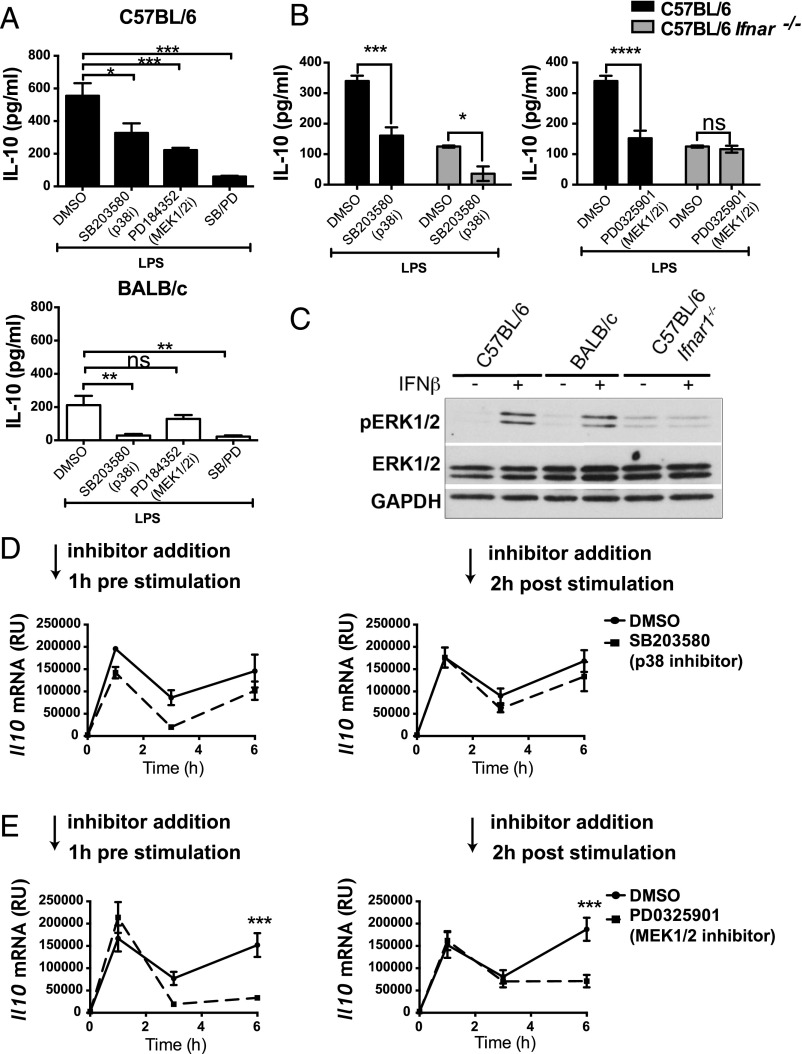
Type I IFN stimulates IL-10 production in C57BL/6 macrophages through the activation of ERK1/2 MAPK. (**A**) C57BL/6 and BALB/c BMDMs were treated with PD184352 and/or SB203580 or DMSO as control 1 h prior to stimulation with LPS for 24 h. IL-10 production was quantified by ELISA. (**B**) C57BL/6 WT and *Ifnar^−/−^* BMDMs were treated with PD0325901 or SB203580 or DMSO as control 1 h prior to stimulation with LPS for 24 h. IL-10 production was quantified by ELISA. (**C**) C57BL/6 WT, *Ifnar1^−/−^*, and BALB/c BMDMs were stimulated with 20 ng/ml IFN-β. Whole-protein extracts were generated and analyzed by Western blot for total and phosphorylated ERK1/2 and GAPDH as loading control. (**D** and **E**) C57BL/6 and BALB/c WT BMDMs were treated with SB203580 or PD0325901 1 h prior to or 2 h after stimulation with LPS. *Il10* mRNA was harvested at indicated times and quantified by qRT-PCR, normalized to *Hprt1* mRNA levels. Graphs show means ± SEM of three to five independent experiments. Western blot shown is representative of three independent experiments. *p < 0.05, **p < 0.01, ***p < 0.001, ns indicates nonsignificant as determined by one-way (A) or two-way ANOVA analysis (Bonferroni multiple comparison test) (B, D, and E).

Our data suggest that type I IFN is an important factor in driving the differential production of IL-10 in C57BL/6 and BALB/c macrophages; however, how type I IFN regulates IL-10 production in macrophages is incompletely understood. ERK1/2 and p38 MAPKs are central regulators of IL-10 in TLR-stimulated C57BL/6 macrophages (reviewed in Ref. 46). We compared the contribution of these MAPKs to IL-10 production in C57BL/6 and BALB/c macrophages by incubating the cells with p38 and MEK1/2 (upstream of ERK1/2) inhibitors. In keeping with previous studies reviewed in Gabryšová et al. (46), we observed reduced IL-10 production in LPS-stimulated C57BL/6 macrophages upon inhibition of p38 or ERK1/2 signaling (Fig. 6A). IL-10 production was further reduced when p38 and ERK1/2 activation was concomitantly blocked (Fig. 6A). In BALB/c macrophages, however, IL-10 production was almost completely abrogated by inhibition of p38 signaling alone, but it was not affected by ERK1/2 inhibition (Fig. 6A). Similarly, in LPS-stimulated C57BL/6 *Ifnar1^−/−^* macrophages, inhibition of p38 signaling significantly reduced IL-10 production, whereas ERK1/2 inhibition had no effect (Fig. 6B). This implied a role for ERK1/2 in the regulation of IL-10 only in the presence of type I IFN. Supporting this, ERK1/2 phosphorylation was induced in C57BL/6 and BALB/c macrophages upon treatment with recombinant IFN-β and this was not observed in C57BL/6 *Ifnar1^−/−^* macrophages, demonstrating the specificity of the IFN-β activation of ERK1/2 (Fig. 6C) and that this was not through activation of any PRR. To formally establish the role of p38 and ERK1/2 in the regulation of IL-10 by type I IFN, we inhibited these MAPKs 1 h before or 2 h after LPS stimulation, with the latter time point representing the peak of autocrine type I IFN signaling (Fig. 3C). Inhibition of p38 prior to LPS stimulation led to a decrease in *Il10* mRNA expression at all time points; however, the type I IFN–dependent second peak of *Il10* mRNA remained present (Fig. 6D). Inhibition of p38 at 2 h poststimulation had no significant effect on *Il10* mRNA levels (Fig. 6D). Inhibition of ERK1/2 signaling at either time point had no effect on the first peak of *Il10* mRNA, whereas it completely blocked the second peak of *Il10* mRNA transcription (Fig. 6E). This result was confirmed using an additional structurally unrelated yet specific MEK1/2 inhibitor (trametinib [from Selleck Chemicals], data not shown). These findings demonstrate a requirement for ERK1/2 in the induction of *Il10* mRNA expression by autocrine type I IFN in C57BL/6 macrophages.

### Reduced levels of IL-1β and IL-12 production observed in C57BL/6 as compared with BALB/c macrophages result from type I IFN action through IL-10–dependent and –independent mechanisms

**FIGURE 7.**
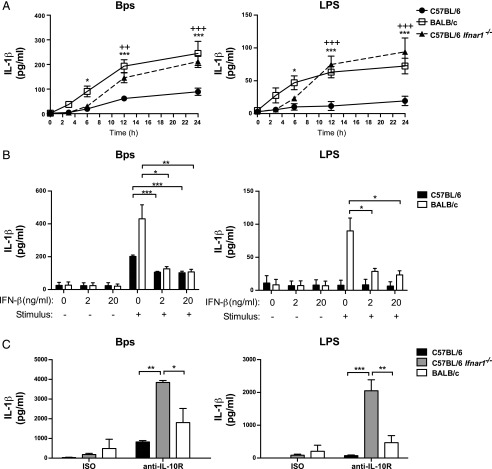
IL-1β production is inhibited in *B. pseudomallei*– and LPS-stimulated C57BL/6 macrophages by type I IFN through IL-10–dependent and –independent mechanisms. (**A**) C57BL/6, BALB/c, and C57BL/6 *Ifnar1^−/−^* BMDMs were stimulated with *B. pseudomallei* or LPS for the indicated times. (**B**) C57BL/6 and BALB/c BMDMs were treated with 2 or 20 ng/ml IFN-β for 2 h prior to stimulation with *B. pseudomallei* or LPS for 24 h. (**C**) C57BL/6, BALB/c, and C57BL/6 *Ifnar1^−/−^* BMDMs were stimulated with *B. pseudomallei* or LPS for 24 h in the presence of anti–IL-10R or isotype control added at the time of stimulation. IL-1β production was quantified by ELISA. Graphs show means ± SEM of two (C) or at least three (A and B) independent experiments. *p < 0.05, **p < 0.01, ***p < 0.001 as determined by one-way (B) or two-way (A and C) ANOVA (*C57BL/6 versus BALB/c, ^+^C57BL/6 versus C57BL/6 *Ifnar1^−/−^*), Bonferroni multiple comparison test.

Because IL-10–mediated inhibition did not fully account for differential IL-1β production in *B. pseudomallei*– or LPS-stimulated C57BL/6 and BALB/c macrophages (Supplemental Fig. 2C), we sought to investigate whether type I IFN had an additional effect on the regulation of IL-1β in these cells. Assessment of IL-1β production in *B. pseudomallei*– and LPS-stimulated C57BL/6 *Ifnar1^−/−^* macrophages revealed significantly elevated IL-1β production relative to C57BL/6 WT macrophages, to levels similar to BALB/c macrophages (Fig. 7A). Indeed, the addition of exogenous IFN-β significantly reduced IL-1β production in both C57BL/6 and BALB/c macrophages, supporting a role for type I IFN in the negative regulation of IL-1β in this system (Fig. 7B). To determine whether type I IFN could negatively regulate IL-1β production through mechanisms other than through the promotion of IL-10 as previously reported (14, 16, 42), IL-1β production was assessed from C57BL/6, BALB/c, and C57BL/6 *Ifnar1^−/−^* macrophages stimulated with *B. pseudomallei* or LPS in the presence of a blocking Ab against the IL-10 receptor (anti-IL-10R) or isotype control Ab (Fig. 7C). The blockade of IL-10 signaling enhanced IL-1β production in all cell types. However, in the presence of anti–IL-10R, IL-1β production was highly elevated in C57BL/6 *Ifnar1^−/−^* macrophages relative to C57BL/6 WT and even BALB/c macrophages (Fig. 7C). Thus, the loss of type I IFN signaling can enhance IL-1β production in the absence of IL-10 signaling, supporting a role for IL-10–independent inhibition of IL-1β production by type I IFN in *B. pseudomallei*– and LPS-stimulated C57BL/6 macrophages. The production of TNF-α in C57BL/6 *Ifnar1^−/−^* macrophages was enhanced relative to C57BL/6 WT in the context of *B. pseudomallei* stimulation, but it was not affected in LPS-stimulated macrophages, implying stimulus-specific regulation of this cytokine by type I IFN (Supplemental Fig. 3E).

**FIGURE 8.**
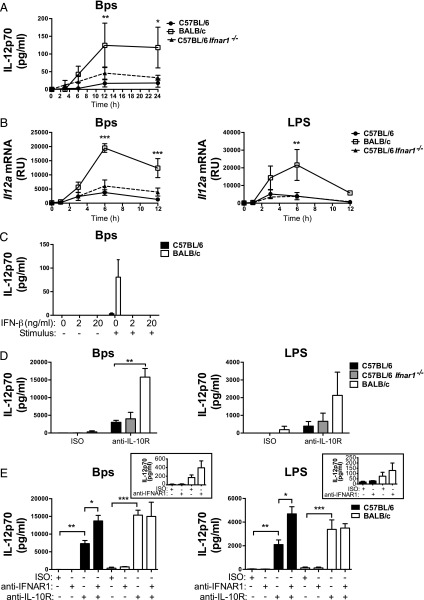
IL-12p70 is negatively regulated by type I IFN and IL-10 in *B. pseudomallei*– and LPS-stimulated C57BL/6 and BALB/c macrophages. (**A** and **B**) C57BL/6, BALB/c, and C57BL/6 *Ifnar1^−/−^* BMDMs were stimulated with *B. pseudomallei* or LPS for the indicated times. (**C**) C57BL/6 and BALB/c BMDMs were treated with 2 or 20 ng/ml IFN-β for 2 h prior to stimulation with *B. pseudomallei* for 24 h. (**D**) C57BL/6, BALB/c, and C57BL/6 *Ifnar1^−/−^* BMDMs were stimulated with *B. pseudomallei* or LPS for 24 h in the presence of anti–IL-10R or isotype control added at the time of stimulation. (**E**) C57BL/6 and BALB/c BMDMs were stimulated with *B. pseudomallei* or LPS for 24 h in the presence of anti-IFNAR1, anti–IL-10R, or isotype control added 2 h prior to stimulation. IL-12p70 production was quantified by ELISA and *Il12a* mRNA expression by qRT-PCR. Graphs show means ± SEM of two (B and D) or at least three (A, C and E) independent experiments. *p < 0.05, **p < 0.01, ***p < 0.001 as determined by two-way ANOVA (Bonferroni multiple comparison test).

Type I IFN has been reported to modulate the levels of IL-12 production in various contexts (15, 16, 18, 19, 33). We observed that IL-12p70 production and *Il12a* expression levels were not different in *B. pseudomallei*– and LPS-stimulated C57BL/6 *Ifnar1^−/−^* macrophages as compared with C57BL/6 WT macrophages (Fig. 8A, 8B). However, the addition of IFN-β abrogated the production of IL-12p70 from *B. pseudomallei*–stimulated cells (no IL-12p70 was detected in LPS-stimulated cells), even in BALB/c macrophages, suggesting that type I IFN does have the capacity to inhibit IL-12p70 production in this system (Fig. 8C). We considered that residual IL-10 production in C57BL/6 *Ifnar1^−/−^* macrophages was preventing the induction of high levels of IL-12p70 production; however, even in the presence of anti–IL-10R, IL-12p70 production was equivalent in C57BL/6 *Ifnar1^−/−^* macrophages compared with C57BL/6 WT macrophages stimulated with *B. pseudomallei* or LPS (Fig. 8D).

Despite a negative regulatory effect of exogenous IFN-β addition on the production of IL-12p70 (Fig. 8C), previous studies have indicated that low-level basal IFN-β production, which gives rise to tonic type I IFN signaling, is required for optimal IL-12 production from innate cells (16, 18). As tonic type I IFN signaling is absent from C57BL/6 *Ifnar1^−/−^* macrophages, we postulated that this may contribute to poor IL-12p70 production, even in the absence of IL-10 signaling (Fig. 8D). Thus, to uncouple tonic type I IFN signaling from PRR-induced autocrine type I IFN signaling, we treated WT C57BL/6 and BALB/c macrophages with anti-IFNAR1 in the presence and absence of anti–IL-10R mAbs (Fig. 8E). Upon treatment with anti-IFNAR1, no effect was observed on IL-12p70 production from *B. pseudomallei*– or LPS-stimulated C57BL/6 macrophages; however, a small increase in IL-12p70 production was observed in BALB/c macrophages (Fig. 8E, inset).

As expected, treatment with anti–IL-10R mAbs significantly increased IL-12p70 production in macrophages from both strains of mice, but IL-12p70 remained higher in BALB/c macrophages. In C57BL/6 macrophages, anti-IFNAR1 treatment in the presence of anti–IL-10R significantly increased IL-12p70 levels relative to anti–IL-10R treatment alone. In contrast, anti-IFNAR1 plus anti–IL-10R mAb–treated BALB/c macrophages did not further enhance IL-12p70 production. Importantly, the level of IL-12p70 production from anti-IFNAR1/anti–IL-10R–treated C57BL/6 and BALB/c macrophages were comparable. Similar results were obtained by treating C57BL/6 *Il10*^−/−^ and BALB/c *Il10*^−/−^ macrophages with anti-IFNAR1 (Supplemental Fig. 3F). These findings demonstrate that deficient IL-12p70 production from C57BL/6 macrophages can be rescued by the elimination of both PRR-induced IL-10 and type I IFN signaling.

## Discussion

The balance of pro- and anti-inflammatory immune responses is essential to ensure effective but safe pathogen clearance. C57BL/6 and BALB/c mice differ significantly in their immune responses during infections and inflammatory diseases (21, 23, 32). A clear example of this is the *B. pseudomallei* infection model where C57BL/6 mice have enhanced resistance compared with BALB/c mice (26–28). The higher production of proinflammatory cytokines in BALB/c mice has been associated with exacerbated *B. pseudomallei*–induced pathology (28–30). However, it is unclear whether this exacerbated pathology is contributed to by decreased control of the pathogen, or by an inability to induce a regulated response. In in vivo settings, signals from multiple cell types are integrated to induce a balanced production of proinflammatory versus anti-inflammatory cytokines such as IL-10 to control pathogens with minimum host damage. This complexity makes the clear dissection of these mechanisms prohibitive in in vivo models. We report in this study, using an in vitro model that allows the examination of these complex phenotypes, that C57BL/6 macrophages produced higher levels of IL-10 but lower levels of TNF-α, IL-12p70, and IL-1β compared with BALB/c macrophages when stimulated with the bacterium *B. pseudomallei*, LPS, or Pam3CSK4. We reveal a central role for autocrine type I IFN in the increased production of IL-10 and increased *Il10* mRNA stability by C57BL/6 macrophages, which is accompanied by increased STAT1 and IRF3 activation. The enhanced and prolonged expression of *Il10* was dependent on type I IFN–induced late ERK1/2 phosphorylation. Conversely, type I IFN suppressed the production of the proinflammatory cytokines IL-12 and IL-1β via IL-10–dependent and –independent mechanisms in *B. pseudomallei*– and LPS-stimulated C57BL/6 macrophages. These findings demonstrate that fundamental differences in type I IFN induction and function in C57BL/6 and BALB/c macrophage responses may contribute to their differential phenotypes.

The differential production of cytokines by macrophages from both mouse strains was not due to different levels of *Tlr4* mRNA expression or protein production, and this was corroborated by similar levels of early p38 and ERK1/2 activation following LPS stimulation. A comparative microarray analysis of temporal gene expression in *B. pseudomallei*–stimulated C57BL/6 and BALB/c macrophages revealed major differences in gene expression, with 790 genes being differentially expressed after 3 h, and strikingly 2246 genes being differentially expressed after 6 h poststimulation. This microarray analysis revealed an unexpected higher expression of type I IFN–responsive genes and type I IFN pathway genes in C57BL/6 macrophages, including *Oas1g*, *Stat1*, *Stat3*, *Irf7*, and *Irf9.* This corresponded with higher IFN-β production in *B. pseudomallei*– and LPS-stimulated C57BL/6 macrophages. Our findings that *B. pseudomallei*–induced IFN-β production was dependent on TLR4 and TRIF, and that IRF3 was more activated in *B. pseudomallei*–stimulated C57BL/6 macrophages, suggest that signaling events affecting the TLR4-TRAM/TRIF-TBK1-IRF3 axis, which is critical for the induction of type I IFN downstream of TLR4 (40), may be responsible for the enhanced production of type I IFN in this strain.

Our findings demonstrating higher levels of expression of type I IFN in C57BL/6 macrophages led us to investigate the potential role of type I IFN in differential production of proinflammatory cytokines and IL-10 in C57BL/6 and BALB/c macrophages. Reduced IL-10 production in *B. pseudomallei*– and LPS-stimulated C57BL/6 *Ifnar1^−/−^* macrophages demonstrated the importance of autocrine type I IFN in maintaining high levels of IL-10 in C57BL/6 macrophages. This is in agreement with previous studies of type I IFN–regulated IL-10 production in TLR4-stimulated macrophages (11, 41, 43). However, our detailed investigation into the mechanisms of *Il10* expression in *B. pseudomallei*– and LPS-stimulated macrophages clearly showed two previously undescribed distinct waves of active *Il10* transcription in C57BL/6 but not BALB/c macrophages, the second of which was completely dependent on type I IFN signaling. We additionally identified a role for autocrine type I IFN in the stabilization of the *Il10* transcript. Complete absence of the type I IFN–dependent second peak of *Il10* mRNA in BALB/c macrophages and the similar level of IL-10 production in BALB/c and C57BL/6 *Ifnar1^−/−^* macrophages stimulated with *B. pseudomallei* and LPS provide evidence for our novel finding that type I IFN drives differential production of IL-10 in C57BL/6 and BALB/c macrophages. This was further supported by our microarray analysis revealing reduced expression of several IL-10 pathway genes in *B. pseudomallei*–stimulated BALB/c and C57BL/6 *Ifnar1^−/−^* macrophages relative to C57BL/6 WT. Thus, although our data suggest that BALB/c macrophages retain responsiveness to type I IFN, we show that the autocrine type I IFN–mediated feed-forward loop, critical for the maintenance of IL-10 production in TLR4-stimulated C57BL/6 macrophages (11, 41, 43), is absent in BALB/c macrophages. Furthermore, the similar levels of IL-10 production in C57BL/6 and BALB/c macrophages stimulated with LPS in the presence of IFN-β suggest that the low level of type I IFN production, as opposed to deficient responsiveness to type I IFN, is a key contributing factor to the reduced levels of IL-10 production observed in BALB/c macrophages.

To date, the mechanisms of IL-10 regulation by type I IFN are incompletely understood. In accordance with the literature (35, 47–51), we show in the present study that IL-10 production by C57BL/6 macrophages in response to TLR ligation required both p38 and ERK1/2 activation, although BALB/c macrophages showed less of a requirement for ERK1/2 activation. Our further findings that inhibition of ERK1/2 signaling also has no effect on IL-10 production in macrophages from C57BL/6 *Ifnar1*^−/−^ mice, and that type I IFN can directly induce ERK1/2 phosphorylation, indicate that ERK1/2 is an important factor in the regulation of IL-10 by type I IFN. In accordance with this, we show prolonged induction of *Il10* mRNA expression to be dependent on ERK1/2 but not p38 activation.

It has been reported that type I IFN requires the production of IL-27 to optimally enhance IL-10 production in C57BL/6 mouse macrophages stimulated with LPS (43). In the present study, we found no role for IL-27 signaling in the production of IL-10 by LPS-stimulated macrophages or the enhancement of IL-10 by IFN-β treatment, although IL-27 production itself was dependent on type I IFN signaling. Our findings thus differ from those of Iyer et al. (43) regarding the ability of IL-27 to induce IL-10 production in C57BL/6 macrophages, perhaps due to differences in BMDM culture or laboratory conditions. However, our results are consistent with previous reports that resting and TLR-stimulated murine macrophages are unresponsive to IL-27 (16, 44) and that IL-27 does not regulate IL-10 production in *M. tuberculosis–*infected macrophages (16). Our findings therefore demonstrate that differential production of IL-10 in C57BL/6 and BALB/c macrophages is due to a type I IFN–dependent but IL-27–independent mechanism.

The assessment of IL-10–deficient C57BL/6 and BALB/c macrophages showed that the differential production of TNF-α, IL-12, and IL-1β in Pam3CSK4-stimulated cells was largely attributable to IL-10. In contrast, only differential TNF-α production was explained by IL-10 in *B. pseudomallei*– and LPS-stimulated C57BL/6 and BALB/c macrophages. IL-1β has previously been reported to be suppressed by type I IFN in macrophages (14, 16, 42). In the absence of type I IFN signaling, we observed an increase in C57BL/6 macrophage IL-1β production to the level of BALB/c macrophages. In keeping with previous reports (14, 16, 42), negative regulation of IL-1β by type I IFN was retained in the absence of IL-10. Indeed, in the absence of both IL-10 and type I IFN signaling, IL-1β production from C57BL/6 *Ifnar1^−/−^* macrophages was higher than that of anti–IL-10R-treated BALB/c macrophages. Thus, C57BL/6 macrophages have a high capacity to produce IL-1β when these two inhibitory loops are removed.

Whereas type I IFN has been shown to negatively regulate macrophage production of TNF-α and IL-1β via mechanisms that are predominantly or only partially dependent on IL-10, respectively (14, 16), it has been reported that type I IFN can both positively and negatively regulate IL-12 production in vitro (15–19). Furthermore, the role for IL-10 in the regulation of IL-12 by type I IFN is not fully understood and may depend on the context (15–17). Despite our finding that treatment with exogenous IFN-β can suppress *B. pseudomallei*–induced IL-12 production, IL-12p70 production was not enhanced in C57BL/6 *Ifnar1^−/−^* macrophages compared with C57BL/6 WT macrophages, even in the presence of anti–IL-10R mAb. We postulated that the failure of C57BL/6 *Ifnar1^−/−^* macrophages to produce high levels of IL-12 was due to the absence of tonic type I IFN signaling, reported to be required for optimal IL-12 production in innate cells (18). Indeed, blockade of type I IFN signaling with the anti-IFNAR1 Ab only 2 h prior to stimulation showed an increase in IL-12p70 protein in C57BL/6 macrophages when IL-10 signaling was concomitantly blocked. The levels of IL-12p70 in C57BL/6 macrophages were similar to those observed in BALB/c macrophages when type I IFN and IL-10 signaling were simultaneously blocked. Collectively, our data show that differential production and function of TLR-induced type I IFN accounts for the differential production of IL-12p70 and IL-1β in C57BL/6 and BALB/c macrophages.

In this study, we demonstrate distinct profiles of pro- and anti-inflammatory cytokine production in C57BL/6 and BALB/c macrophages and provide mechanisms to account for these differences. C57BL/6 macrophages, in response to TLR4 ligation, produced higher levels of type I IFN accompanied by increased induction of type I IFN–inducible genes and STAT1 activation, as compared with BALB/c macrophages. Type I IFN was found to induce increased and sustained *Il10* mRNA expression via an ERK1/2-dependent pathway, demonstrating the importance of temporal regulation of cytokine gene expression. Additionally, type I IFN increased *Il10* mRNA stability. Collectively, these effects of type I IFN resulted in the increased IL-10 protein production observed in C57BL/6 macrophages. Type I IFN regulated IL-12p70 and IL-1β production via IL-10–dependent and IL-10–independent mechanisms, with both accounting for the differential production of these proinflammatory cytokines by C57BL/6 and BALB/c macrophages. This work highlights the complex role of type I IFN in the regulation of innate immune responses, and it further suggests that the extent of type I IFN–mediated activity may differ according to the genetic background of the host. Our findings emphasize the fact that the C57BL/6 genetic strain of mouse, commonly used in immunological studies, may not reflect the true breadth of immunoregulatory mechanisms operating in TLR-stimulated macrophages, or in broader immunological contexts, and they support the dissection of complex molecular mechanisms of cytokine gene regulation in model systems.

## Supplementary Material

Data Supplement
