# Supplementary material for: Differential Production of Type I IFN Determines the Reciprocal Levels of IL-10 and Proinflammatory Cytokines Produced by C57BL/6 and BALB/c Macrophages
Source: J Immunol. 2016 Aug 22;197(7):2838–53. doi: 10.4049/jimmunol.1501923 (PMC5026030; doi:10.4049/jimmunol.1501923)
Supplement: Data Supplement [file JI_1501923.zip › JI_1501923_Supplemental_Material_1.pdf]

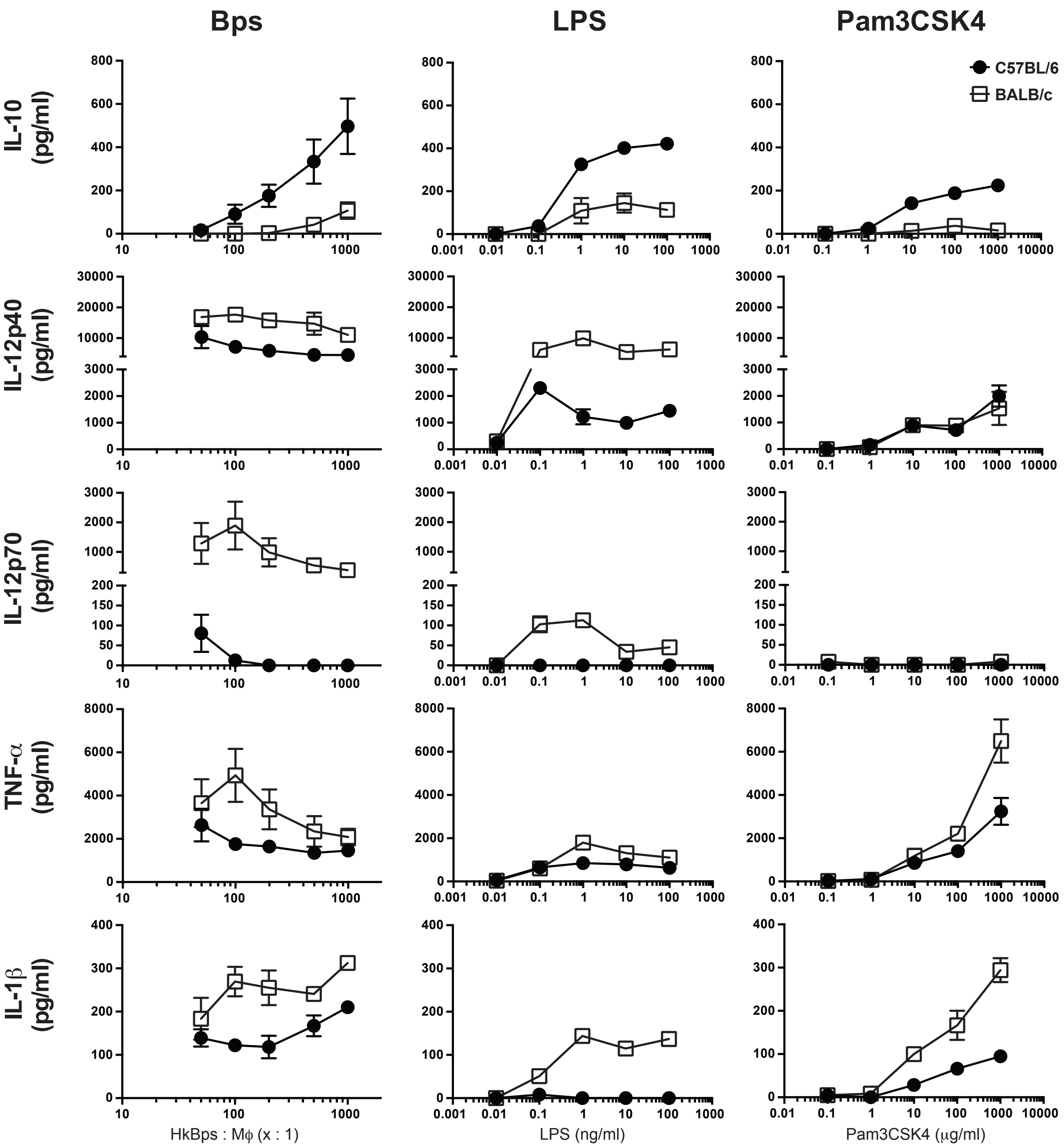

SUPPLEMENTARY FIGURE 1. C57BL/6 macrophages produce higher levels of IL-10 whereas BALB/c macrophages produce higher levels of proinflammatory cytokines across the dose response to *B. pseudomallei*, LPS and Pam3CSK4. C57BL/6 and BALB/c BMDMs were stimulated with the indicated doses of *Bps*, LPS or Pam3CSK4 for 24 h. Cytokine levels in supernatants were determined by ELISA. Graphs show means ±SD of triplicate cultures. Representative of 2 independent experiments.

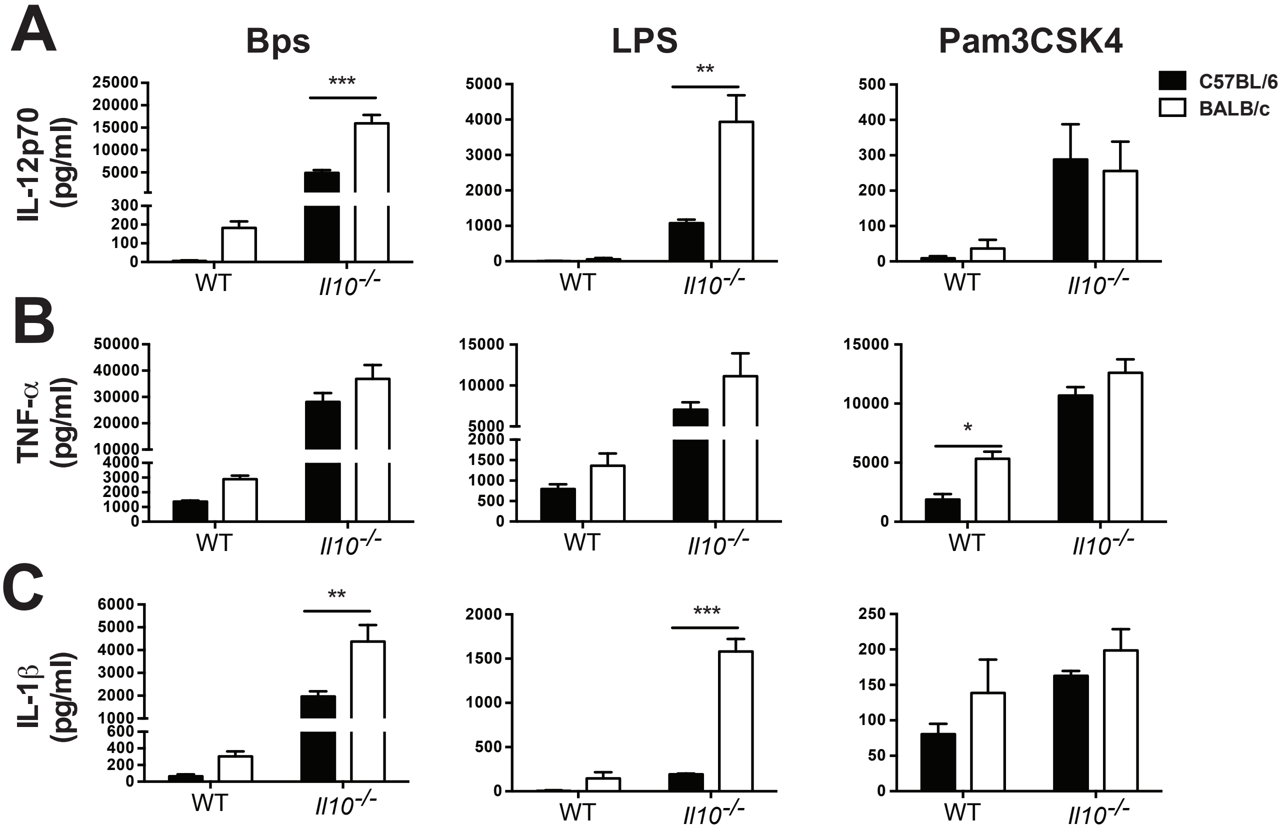

SUPPLEMENTARY FIGURE 2. Reduced IL-12p70 and IL-1β production in LPS and *B. pseudomallei* stimulated C57BL/6 macrophages is not fully accounted for by IL-10 production. C57BL/6 WT, BALB/c WT, C57BL/6 *Il10*$^{-/-}$ and BALB/c *Il10*$^{-/-}$ BMDMs were stimulated with *Bps*, LPS or Pam3CSK4 for 24 h. IL-12p70 (A), TNF-α (B) and IL-1β (C) levels were determined by ELISA. Graphs show means ±SEM of 3 - 4 independent experiments.*p<0.05, **p<0.01, ***p<0.001 as determined by two-way ANOVA (Bonferroni's multiple comparison test).

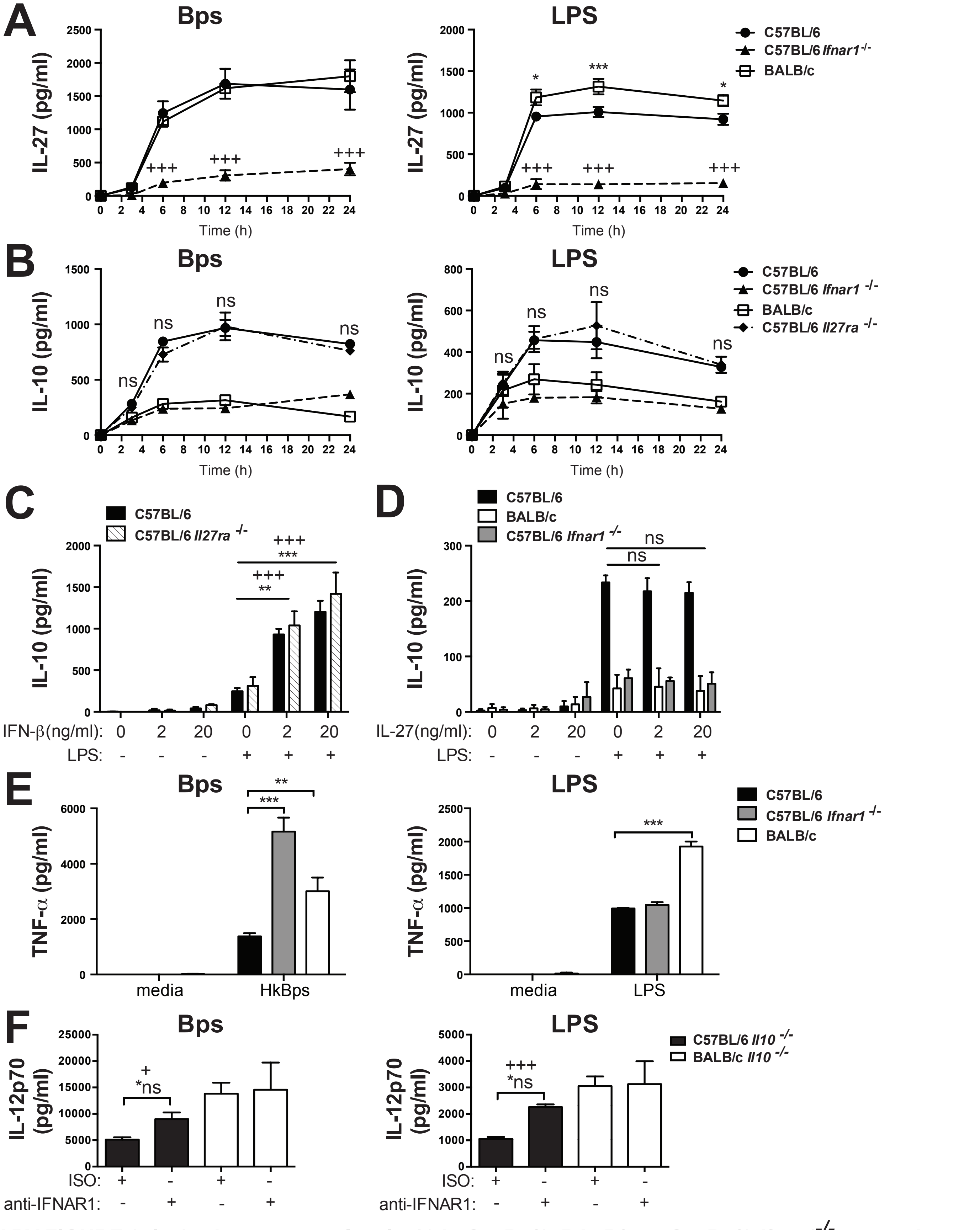

SUPPLEMENTARY FIGURE 3. IL-27 does not regulate IL-10 in C57BL/6, BALB/c or C57BL/6 *Ifnar1*$^{-/-}$ macrophages stimulated with *B. pseudomallei* or LPS. (A, B) C57BL/6, BALB/c, C57BL/6 *Ifnar1*$^{-/-}$ and C57BL/6 *Il27ra*$^{-/-}$ BMDMs were stimulated with *Bps* or LPS for the indicated times. Statistics were determined by two-way ANOVA (Bonferroni's multiple comparison test) and show significance of (A) *C57BL/6 vs. BALB/c or +C57BL/6 vs. C57BL/6 *Ifnar1*$^{-/-}$ or (B) C57BL/6 vs. C57BL/6 I*l27ra*$^{-/-}$ for each time-point. (C) C57BL/6 and C57BL/6 *Il27ra*$^{-/-}$ BMDMs were stimulated for 24 h with LPS in the presence or absence of 2 h pre-incubation with IFN-β. Statistics compare LPS alone to LPS with 2 or 20 ng/ml IFN-β for *C57BL/6 or +C57BL/6 *Il27ra*$^{-/-}$ BMDMs as determined by two-way ANOVA (Bonferroni's multiple comparison test). (D) C57BL/6, BALB/c and C57BL/6 *Ifnar1*$^{-/-}$ BMDMs were stimulated for 24 h with LPS in the presence or absence of 2 h pre-incubation with IL-27. (E) C57BL/6, BALB/c and C57BL/6 *Ifnar1*$^{-/-}$ BMDMs were stimulated with *Bps* or LPS for 24 h. (F) C57BL/6 *Il10*$^{-/-}$ and BALB/c *Il10*$^{-/-}$ BMDMs were stimulated with *Bps* or LPS for 24 h in the presence of anti-IFNAR1 or isotype control added 2 h prior to stimulation. Statistics were determined by two-way ANOVA (*) (Bonferroni's multiple comparison test) or Student's t-test (+). Cytokine levels in supernatants were determined by ELISA. Graphs show means ±SEM of 2-3 independent experiments. *p<0.05, **p<0.01, ***p<0.001

Supplementary Table 1
Genes and related log FC associated with IPA canonical payhways

Genes in the Interferon Signaling network

Symbol	Log FC C57BL/6-Bps vs. BALB/c-BPS (3h)	Log FC C57BL/6-Bps vs. BALB/c-BPS (6h)
G1P2	N/A	0.29
IFI35	1.33	0.61
IFIT1	2.53	2.77
IFIT3	N/A	1.33
IFITM2	0.58	N/A
IFNB1	N/A	-0.92
IFNGR2	0.62	0.92
IFNγRα	N/A	1.66
IRF9	N/A	0.41
NF-κBp65	N/A	0.33
OAS1	0.94	0.60
PIAS1	0.82	0.79
STAT1	1.01	0.55
STAT2	0.86	0.54
TAP1	1.22	0.72
TYK2	0.79	N/A

Genes in the JAK/Stat Signaling network

Symbol	Log FC C57BL/6-Bps vs. BALB/c-BPS (3h)	Log FC C57BL/6-Bps vs. BALB/c-BPS (6h)
c-FOS	N/A	1.99
c-JUN	2.03	N/A
CEBPÎ²	N/A	0.69
CIS	N/A	-1.22
GNAQ	0.78	N/A
KRAS	N/A	-1.60
MRAS	N/A	-1.70
NFKB2	2.97	3.51
PIAS1	0.82	0.79
PIAS3	-0.92	-0.56
PIK3CD	1.56	1.90
PIK3CG	1.10	N/A
RELA	N/A	0.33
RRAS2	N/A	-3.44
SOCS4	1.39	N/A
STAT1	1.01	0.55
STAT2	0.86	0.54
STAT3	1.76	1.39
STAT4	2.47	1.72
TYK2	0.79	N/A

Genes in the Activation of IRF by Cytosolic Pattern Recognition Receptors network

Symbol	Log FC C57BL/6-Bps vs. BALB/c-BPS (3h)	Log FC C57BL/6-Bps vs. BALB/c-BPS (6h)
ADAR1	1.21	0.55
c-Jun	2.03	N/A
CD-40	N/A	2.84
DAI	N/A	0.66
DDX58	1.24	N/A
DHX58	1.52	0.91
IFNB1	N/A	-0.92
IKBKB	N/A	0.93
IKBKE	N/A	0.85
IL-10	N/A	3.29
IRF-7	N/A	0.65
IRF9	N/A	0.41
ISG-15	N/A	0.29
ISG-54	1.10	1.13
MAP2K4	1.30	1.08
NFKB2	2.97	3.51
NFKBIA	N/A	0.21
p300/CBP	N/A	0.94
p65NFκB	N/A	0.33
STAT1	1.01	0.55
STAT2	0.86	0.54
TANK	N/A	0.56
TNFα	N/A	-0.14

Genes in the Role of JAK1, JAK2 and TYK2 in Interferon Signaling network

Symbol	Log FC C57BL/6-Bps vs. BALB/c-BPS (3h)	Log FC C57BL/6-Bps vs. BALB/c-BPS (6h)
IFNB1	N/A	-0.92
IFNGR1	N/A	1.66
IFNGR2	0.62	0.92
NFKB2	2.97	3.51
RELA	N/A	0.33
STAT1	1.01	0.55
STAT2	0.86	0.54
STAT3	1.76	1.39
TYK2	0.79	N/A

Genes in the Role of PKR in Interferon Induction and Antiviral Response network

Symbol	Log FC C57BL/6-Bps vs. BALB/c-BPS (3h)	Log FC C57BL/6-Bps vs. BALB/c-BPS (6h)
APAF-1	1.26	0.64
BID	0.47	0.77
CASP8	N/A	0.88
CASP9	N/A	-0.29
FcγRI	N/A	1.24
IFNβ	N/A	-0.92
IKBKB	N/A	0.93
IKBKE	N/A	0.85
MAP2K3	0.73	0.27
NFKB2	2.97	3.51
NFKBIA	N/A	0.21
p38MAPK	N/A	-0.77
RELA	N/A	0.33
STAT1	1.01	0.55
TNF-α	N/A	-0.14
TNFR1	1.44	1.23
TRAF2	N/A	0.42

Genes in the Type I Diabetes Mellitus Signaling network

Symbol	Log FC C57BL/6-Bps vs. BALB/c-BPS (3h)	Log FC C57BL/6-Bps vs. BALB/c-BPS (6h)
APAF1	1.26	0.64
BID	0.47	0.77
CASP8	N/A	0.88
CASP9	N/A	-0.29
CD86	1.97	1.40
CPE	N/A	-1.28
FAS	N/A	5.19
HLA-A	-3.94	-10.48
HLA-DMA	N/A	-0.87
HLA-DQB1	N/A	6.63
HLA-DRA	-5.16	-5.58
HLA-E	0.54	N/A
IFNγR1	N/A	1.66
IFNγR2	0.62	0.92
IKBKB	N/A	0.93
IKBKE	N/A	0.85
IL12A	N/A	-4.40
MAP2K3	0.73	0.27
MAP2K4	1.30	1.08
MAPK14	N/A	-0.77
MYD88	1.16	0.54
NFKB2	2.97	3.51
NFKBIA	N/A	0.21
PIAS1	0.82	0.79
RELA	N/A	0.33
SOCS4	1.39	N/A
STAT1	1.01	0.55
TNF-α	N/A	-0.14
TNFRSF1A	1.44	1.23
TNFRSF1B	N/A	0.62
TRAF2	N/A	0.42

Genes in the Antigen Presentation Pathway network

Symbol	Log FC C57BL/6-Bps vs. BALB/c-BPS (3h)	Log FC C57BL/6-Bps vs. BALB/c-BPS (6h)
CALR	N/A	-0.47
CLIP	1.28	0.45
HLA-A	-3.94	-10.48
HLA-DMA	N/A	-0.87
HLA-DRA	-5.16	-5.58
HLA-E	0.54	N/A
LMP2	N/A	5.03
LMPX	N/A	1.43
MHCI-β	0.76	N/A
NLRC5	1.56	1.06
TAP1	1.22	0.72
TAP2	N/A	0.39
TPN	1.66	1.53

Genes in the Dendritic Cell Maturation network

Symbol	Log FC C57BL/6-Bps vs. BALB/c-BPS (3h)	Log FC C57BL/6-Bps vs. BALB/c-BPS (6h)
ATF4	N/A	4.43
B2M	0.76	N/A
CD40	N/A	2.84
CD86	1.97	1.40
CREBBP	N/A	0.94
FCGR1A	N/A	1.24
FCGR2A	-0.24	-0.24
FCGR3A/FCGR3B	1.69	1.41
FSCN1	N/A	-0.99
GM-CSF	N/A	-3.41
HLA-A	-3.94	-10.48
HLA-DMA	N/A	-0.87
HLA-DQB1	N/A	6.63
HLA-DRA	-5.16	-5.58
IFNB1	N/A	-0.92
IKBKB	N/A	0.93
IKBKE	N/A	0.85
IL-10	N/A	3.29
IL-15	N/A	0.87
IL-23p19	N/A	-1.30
IL12A	N/A	-4.40
IL1A	N/A	-1.29
IL1RN	N/A	1.07
LTB	-2.19	-3.45
MAP2K4	1.30	1.08
MAPK14	N/A	-0.77
MYD88	1.16	0.54
NFKBIA	N/A	0.21
NFκBp52	2.97	3.51
PIK3CD	1.56	1.90
PIK3CG	1.10	N/A
PLCB2	2.63	1.69
PLCL2	1.03	N/A
RELA	N/A	0.33
STAT1	1.01	0.55
STAT2	0.86	0.54
STAT4	2.47	1.72
TLR2	N/A	-0.14
TLR4	N/A	1.61
TNF-α	N/A	-0.14
TNFRSF1A	1.44	1.23
TNFRSF1B	N/A	0.62

Genes in the OX40 Signaling Pathway network

Symbol	Log FC C57BL/6-Bps vs. BALB/c-BPS (3h)	Log FC C57BL/6-Bps vs. BALB/c-BPS (6h)
B2M	0.76	N/A
H2-K2/H2-Q9	N/A	1.88
H2-M2	N/A	0.56
H2-Q8	-1.35	-1.56
H2-T10	4.93	4.97
H2-T24	N/A	-0.32
HLA-A	-3.94	-10.48
HLA-DMA	N/A	-0.87
HLA-DQB1	N/A	6.63
HLA-DRA	-5.16	-5.58
HLA-E	0.54	N/A
JUN	2.03	N/A
MAP2K4	1.30	1.08
NFKB2	2.97	3.51
NFKBIA	N/A	0.21
RELA	N/A	0.33
TRAF2	N/A	0.42

Genes in the Pancreatic Adenocarcinoma Signaling network

Symbol	Log FC C57BL/6-Bps vs. BALB/c-BPS (3h)	Log FC C57BL/6-Bps vs. BALB/c-BPS (6h)
CASP9	N/A	-0.29
CDKN1B	1.87	N/A
CyclinD1	N/A	2.14
E2F2	N/A	0.18
E2F6	N/A	-1.40
HMOX1	N/A	1.05
K-Ras	N/A	-1.60
MAP2K4	1.30	1.08
MDM2	1.19	0.70
MMP9	-1.49	-2.80
NFKB2	2.97	3.51
PIK3CD	1.56	1.90
PIK3CG	1.10	N/A
PLD1	N/A	0.51
RELA	N/A	0.33
SMAD2	2.04	1.85
SMAD3	1.34	N/A
STAT1	1.01	0.55
STAT3	1.76	1.39
SURVIVIN	N/A	-0.83
TGFβR2	N/A	-1.56
TYK2	0.79	N/A
VEGFA	1.54	N/A

Genes in the phagosome maturation network

Symbol	Log FC C57BL/6-Bps vs. BALB/c-BPS (3h)	Log FC C57BL/6-Bps vs. BALB/c-BPS (6h)
ATP6V0A1	N/A	-0.32
ATP6V0B	1.05	0.59
ATP6V0E2	N/A	-2.46
B2M	0.76	N/A
CALR	N/A	-0.47
DCTN4	N/A	0.78
Dync1i2	N/A	0.97
DYNLRB1	0.83	0.44
HLA-A	-3.94	-10.48
HLA-DRA	-5.16	-5.58
LAMP2	0.55	0.81
NAPB	N/A	0.01
PRDX2	N/A	0.61
RAB7B	1.17	4.11
TAP1	1.22	0.72
TUBB2B	-0.55	-2.36
TUBG1	N/A	-1.09
VAMP3	0.90	N/A
VPS33A	-0.43	-0.74
VPS37B	1.24	N/A
VTI1A	N/A	1.34
VTI1B	N/A	2.89
YKT6	0.88	N/A

Genes in the Death Receptor Signaling network

Symbol	Log FC C57BL/6-Bps vs. BALB/c-BPS (3h)	Log FC C57BL/6-Bps vs. BALB/c-BPS (6h)
APAF1	1.26	0.64
BID	0.47	0.77
CASP9	N/A	-0.29
CASP8	N/A	0.88
DAXX	1.17	0.81
DR6	N/A	-0.47
FAS	N/A	5.19
HtrA2	N/A	-0.57
IKBKB	N/A	0.93
IKBKE	N/A	0.85
MAP2K4	1.30	1.08
NFKB2	2.97	3.51
NFKBIA	N/A	0.21
PARP12	N/A	0.68
PARP14	0.96	0.59
PARP2	N/A	0.18
PARP3	N/A	2.00
PARP4	N/A	0.71
PARP8	N/A	3.78
PARP9	N/A	0.72
RELA	N/A	0.33
TANK	N/A	0.56
TNF-R2	N/A	0.62
TNF-α	N/A	-0.14
TNFRSF1A	1.44	1.23
TRAF2	N/A	0.42
ZC3HAV1	N/A	0.35

Genes in the Crosstalk between Dendritic Cells and Natural Killer Cells network

Symbol	Log FC C57BL/6-Bps vs. BALB/c-BPS (3h)	Log FC C57BL/6-Bps vs. BALB/c-BPS (6h)
CD40	N/A	2.84
CD69	N/A	0.61
CD86	1.97	1.40
CSF2RB	N/A	0.65
Fas	N/A	5.19
FSCN1	N/A	-0.99
GM-CSF	N/A	-3.41
HLA-A	-3.94	-10.48
HLA-DRA	-5.16	-5.58
HLA-E	0.54	N/A
IFNB1	N/A	-0.92
IL-15	N/A	0.87
IL12A	N/A	-4.40
IL15RA	1.67	1.10
IL3RA	1.58	1.65
LFA-1	N/A	-1.00
LTB	-2.19	-3.45
MICB	1.07	N/A
Nectin2	N/A	-0.92
NFKB2	2.97	3.51
RELA	N/A	0.33
TLR4	N/A	1.61
TLR7	N/A	0.63
TNF-α	N/A	-0.14
TNFR2	N/A	0.62

Genes in the Role of Macrophages, Fibroblasts and Endothelial Cells in Rheumatoid Arthritis network

Symbol	Log FC C57BL/6-Bps vs. BALB/c-BPS (3h)	Log FC C57BL/6-Bps vs. BALB/c-BPS (6h)
ADAMTS4	N/A	-2.48
ATF4	N/A	4.43
c-Fos	N/A	1.99
c-Jun	2.03	N/A
C5AR	N/A	0.25
CEBPB	N/A	0.69
CREBBP	N/A	0.94
CyclinD1	N/A	2.14
DAMM1	1.66	N/A
DVL	N/A	-1.11
FcγRIIIa	1.69	1.41
FCγR1	N/A	1.24
FN1	N/A	-0.77
FZD7	N/A	1.02
GM-CSF	N/A	-3.41
GNAQ	0.78	N/A
IKBKB	N/A	0.93
IKBKE	N/A	0.85
IL-10	N/A	3.29
IL-15	N/A	0.87
IL-6R	N/A	1.00
IL1A	N/A	-1.29
IL1RN	N/A	1.07
IRAK3	N/A	0.23
KRAS	N/A	-1.60
LTB	-2.19	-3.45
MAP2K3	0.73	0.27
MAP2K4	1.30	1.08
MCP-1	N/A	0.61
MRAS	N/A	-1.70
MYD88	1.16	0.54
NFATC3	N/A	0.67
NFKBIA	N/A	0.21
NLK	N/A	-1.21
OSM	N/A	-0.64
p38 MAPKα	N/A	-0.77
PDGFB	N/A	0.78
PIK3CD	1.56	1.90
PIK3CG	1.10	N/A
PLCB2	2.63	1.69
PLCL2	1.03	N/A
PPP3R1	N/A	-0.37
PRKD3	N/A	-0.94
RANTES	N/A	0.22
RELA	N/A	0.33
RRAS2	N/A	-3.44
RYK	N/A	-3.28
Src	N/A	-0.80
STAT3	1.76	1.39
TCF4	0.85	1.00
TLR2	N/A	-0.14
TLR4	N/A	1.61
TLR6	N/A	-0.53
TLR7	N/A	0.63
TNF	N/A	-0.14
TNFRSF1A	1.44	1.23
TNFRSF1B	N/A	0.62
TRAF1	N/A	0.40
TRAF2	N/A	0.42
VCAM-1	N/A	-1.31
VEGFA	1.54	N/A

Genes in the Role of Pattern Recognition Receptors in Recognition of Bacteria and Viruses network

Symbol	Log FC C57BL/6-Bps vs. BALB/c-BPS (3h)	Log FC C57BL/6-Bps vs. BALB/c-BPS (6h)
C1QB	N/A	3.17
C3	N/A	0.35
C5aR	N/A	0.25
Casp1	N/A	0.48
CSF2	N/A	-3.41
DECTIN-1	N/A	-0.89
DECTIN-2	N/A	0.52
IFNB1	N/A	-0.92
IL-10	N/A	3.29
IL12A	N/A	-4.40
IL1A	N/A	-1.29
IRF-7	N/A	0.65
MAP2K4	1.30	1.08
MYD88	1.16	0.54
NFKB2	2.97	3.51
NOD1	N/A	1.42
NOD2	-1.50	-1.79
OAS1	0.94	0.60
OAS3	1.46	1.01
OSM	N/A	-0.64
PIK3CD	1.56	1.90
PIK3CG	1.10	N/A
PRKD3	N/A	-0.94
PTX3	N/A	4.34
RANTES	N/A	0.22
RELA	N/A	0.33
RIG-1	1.24	N/A
TLR2	N/A	-0.14
TLR4	N/A	1.61
TLR6	N/A	-0.53
TLR7	N/A	0.63
TNF-α	N/A	-0.14

Genes in the Communication between Innate and Adaptive Immune Cells network

Symbol	Log FC C57BL/6-Bps vs. BALB/c-BPS (3h)	Log FC C57BL/6-Bps vs. BALB/c-BPS (6h)
B2M	0.76	N/A
CCL3L3	N/A	0.25
CD40	N/A	2.84
CD86	1.97	1.40
CD8B	-0.76	-1.76
GM-CSF	N/A	-3.41
HLA-A	-3.94	-10.48
HLA-DRA	-5.16	-5.58
HLA-E	0.54	N/A
IFNB1	N/A	-0.92
IL-10	N/A	3.29
IL-15	N/A	0.87
IL12A	N/A	-4.40
IL1A	N/A	-1.29
IL1RN	N/A	1.07
IP-10	N/A	3.64
RANTES	N/A	0.22
TLR2	N/A	-0.14
TLR4	N/A	1.61
TLR6	N/A	-0.53
TLR7	N/A	0.63
TNF-α	N/A	-0.14
